# Modulation of metabolic, inflammatory and fibrotic pathways by semaglutide in metabolic dysfunction-associated steatohepatitis

**DOI:** 10.1038/s41591-025-03799-0

**Published:** 2025-07-21

**Authors:** Maximilian Jara, Jenny Norlin, Mette Skalshøi Kjær, Kasper Almholt, Kristian M. Bendtsen, Elisabetta Bugianesi, Kenneth Cusi, Elisabeth D. Galsgaard, Milan Geybels, Lise L. Gluud, Lea M. Harder, Rohit Loomba, Gianluca Mazzoni, Philip N. Newsome, Louise M. Nitze, Mads S. Palle, Vlad Ratziu, Anne-Sophie Sejling, Vincent W.-S. Wong, Quentin M. Anstee, Lotte B. Knudsen

**Affiliations:** 1 Novo Nordisk, Clinical Development, Søborg, Denmark; 2 Novo Nordisk, Research & Early Development, Måløv, Denmark; 3 Division of Gastroenterology, Department of Medical Sciences, University of Torino, Torino, Italy; 4 Division of Endocrinology, Diabetes, and Metabolism, University of Florida, Gainesville, FL USA; 5 Novo Nordisk, Data Science, Søborg, Denmark; 6 Gastro Unit, Copenhagen University Hospital Hvidovre, Hvidovre, Denmark and Department of Medicine, University of Copenhagen, Copenhagen, Denmark; 7 Radcliffe Department of Medicine, Oxford University, Oxford, UK; 8 Roger Williams Institute of Liver Studies, School of Immunobiology and Microbial Sciences, Faculty of Life Sciences & Medicine, King’s College London and King’s College Hospital, London, UK; 9 Sorbonne University, ICAN – Institute for Cardiometabolism and Nutrition, Hôpital Pitié Salpêtrière, Paris, France; 10 INSERM UMRS 1138 CRC, Paris, France; 11 Department of Medicine and Therapeutics, The Chinese University of Hong Kong, Hong Kong, China; 12 Translational & Clinical Research Institute, Faculty of Medical Sciences, Newcastle University, Newcastle upon Tyne, UK; 13 Newcastle NIHR Biomedical Research Centre, Newcastle upon Tyne Hospitals NHS Trust, Newcastle upon Tyne, UK

**Keywords:** Metabolic disorders, Proteomic analysis

## Abstract

Metabolic dysfunction-associated steatohepatitis (MASH) is a chronic liver disease strongly associated with cardiometabolic risk factors. Semaglutide, a glucagon-like peptide-1 receptor agonist, improves liver histology in MASH, but the underlying signals and pathways driving semaglutide-induced MASH resolution are not well understood. Here we show that, in two preclinical MASH models, semaglutide improved histological markers of fibrosis and inflammation and reduced hepatic expression of fibrosis-related and inflammation-related gene pathways. Aptamer-based proteomic analyses of serum samples from patients with MASH in a clinical trial identified 72 proteins significantly associated with MASH resolution and semaglutide treatment, with most related to metabolism and several implicated in fibrosis and inflammation. An independent real-world cohort verified the pathophysiological relevance of this signature, showing that the same 72 proteins are differentially expressed in patients with MASH relative to healthy individuals. Taken together, these data suggest that semaglutide may revert the circulating proteome associated with MASH to the proteomic pattern observed in healthy individuals.

## Abstract (web summary)

A combination of animal studies, data from a clinical trial and insights from an observational cohort suggest that, in individuals with metabolic dysfunction-associated steatohepatitis, semaglutide improves liver histology by reverting patient proteomes to better mimic the proteome of individuals without the disease.

## Main

Semaglutide is a glucagon-like peptide-1 receptor agonist (GLP-1RA) approved for the treatment of patients with type 2 diabetes (T2D) who are not satisfactorily controlled through diet and exercise and other glucose-lowering medications^1^ and for weight management in adults with overweight or obesity^2^. MASH is a severe form of metabolic dysfunction-associated steatotic liver disease (MASLD) and is associated with chronic inflammation that causes progressive fibrosis, which may lead to cirrhosis and/or hepatocellular carcinoma (HCC)^3,4^. In a phase 2 trial (NCT02970942) of 320 individuals with biopsy-confirmed MASH, semaglutide treatment significantly increased the number of patients with resolution of MASH versus placebo^5^. In an interim analysis of the ongoing phase 3 ‘Effect of Semaglutide in Subjects with Non-cirrhotic Non-alcoholic Steatohepatitis’ (ESSENCE) trial (NCT04822181), once-weekly semaglutide 2.4 mg demonstrated superiority versus placebo for improvement of histological activity and fibrosis in participants with MASH and moderate to advanced liver fibrosis^6^. In the present study, we investigated signals and pathways by which semaglutide may exert its beneficial effects in MASH, with relation to body weight loss and other potential mechanisms of histological improvement.

## Results

### Metabolic factors and histologic efficacy of semaglutide

**Fig. 1:**
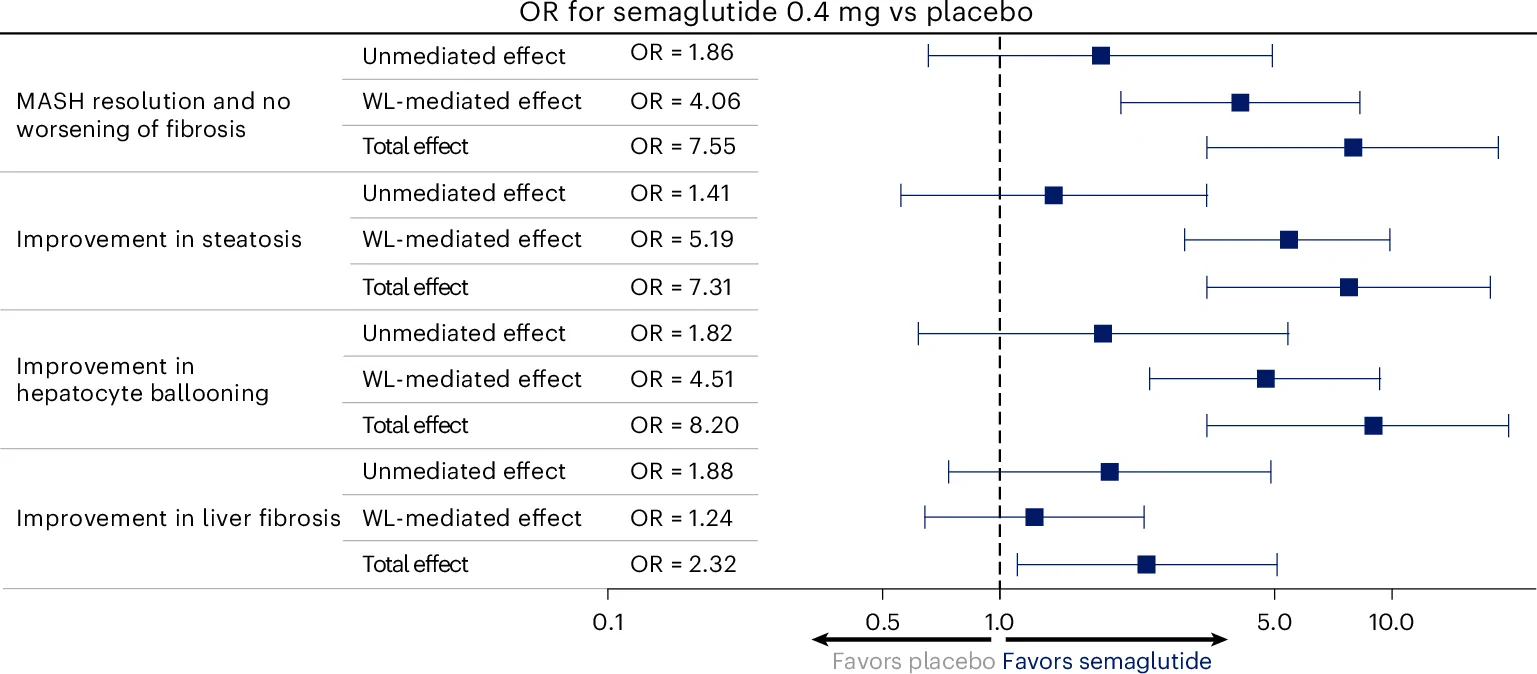
Mediated (WL) and unmediated (WL-independent) treatment effect on histological improvement with semaglutide versus placebo. Data were based on complete-case on-treatment measurements (*N* = 249) for histological parameters that showed a statistically significant effect of semaglutide. Data are shown as odds ratios (ORs) (center point) and 95% CIs. Mediator was WL at weeks 4, 12, 20, 28, 36, 44, 52, 62 and 72. Baseline confounders were age, T2D, fibrosis stage, body weight and gender. WL, weight loss. Source data

**Fig. 2:**
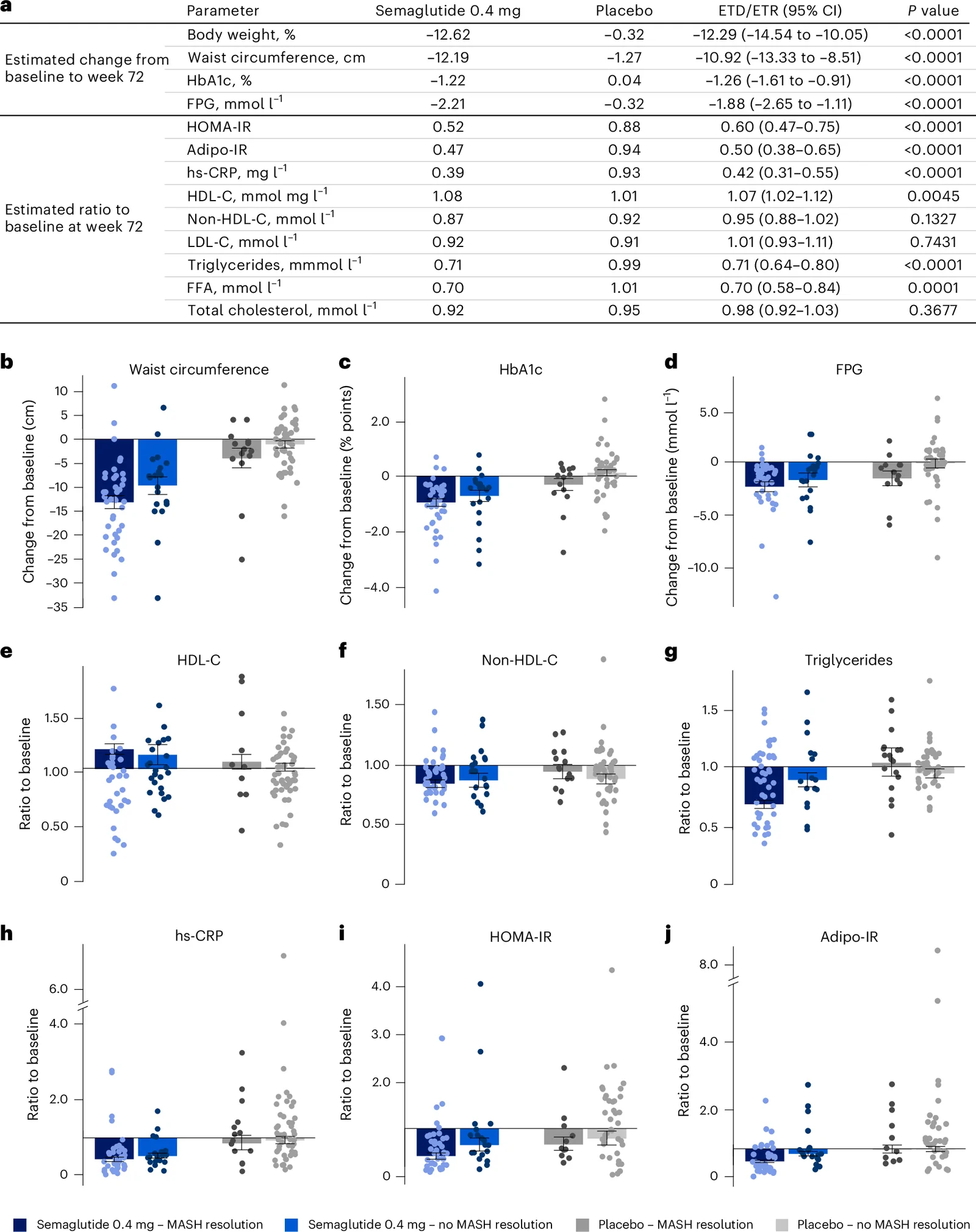
Improvements in metabolic factors are correlated with MASH resolution without worsening of fibrosis. **a**, On-treatment observations using an MMRM. Data are partially presented in the primary publication. Differences between semaglutide and placebo were assessed using a two-sided *t*-test. HbA1c and FPG are reported in patients with T2D only, and HOMA-IR and Adipo-IR (fasting plasma insulin × FFA) are reported in patients not treated with insulin at baseline. **b**–**j**, Data for change from baseline are mean ± s.e.m. and, for ratio to baseline, geometric mean ± s.e.m. calculated on a log scale and then back-transformed. Data are from the on-treatment observation period for individuals with available data (complete-case). **b**, Waist circumference (*n* = 40, *n* = 20, *n* = 14 and *n* = 49, respectively). **c**, HbA1c in individuals with and without T2D (*n* = 39, *n* = 20, *n* = 14 and *n* = 48, respectively). **d**, FPG in individuals both with and without T2D (*n* = 40, *n* = 20, *n* = 14 and *n* = 48, respectively). **e**, HDL-C (*n* = 40, *n* = 20, *n* = 14 and *n* = 48, respectively). **f**, Non-HDL-C (*n* = 40, *n* = 20, *n* = 14 and *n* = 48, respectively). **g**, Triglycerides (*n* = 40, *n* = 20, *n* = 14 and *n* = 48, respectively). **h**, hs-CRP (*n* = 40, *n* = 20, *n* = 14 and *n* = 48, respectively). **i**, HOMA-IR in individuals not treated with insulin at baseline (*n* = 39, *n* = 19, *n* = 11 and *n* = 40, respectively). **j**, Adipo-IR in individuals not treated with insulin at baseline (*n* = 39, *n* = 19, *n* = 11 and *n* = 39, respectively). Adipo-IR, adipose tissue insulin resistance (fasting plasma insulin × FFA); ETD, estimated treatment difference; ETR, estimated treatment ratio; FFA, free fatty acids; FPG, fasting plasma glucose; HbA1c, glycated hemoglobin; HDL-C, high-density lipoprotein cholesterol; HOMA-IR, homeostatic model assessment of insulin resistance; hs**-**CRP, high-sensitivity C-reactive protein; LDL-C, low-density lipoprotein cholesterol; s.e.m., standard error of the mean. Source data

**Extended Data Fig. 1:**
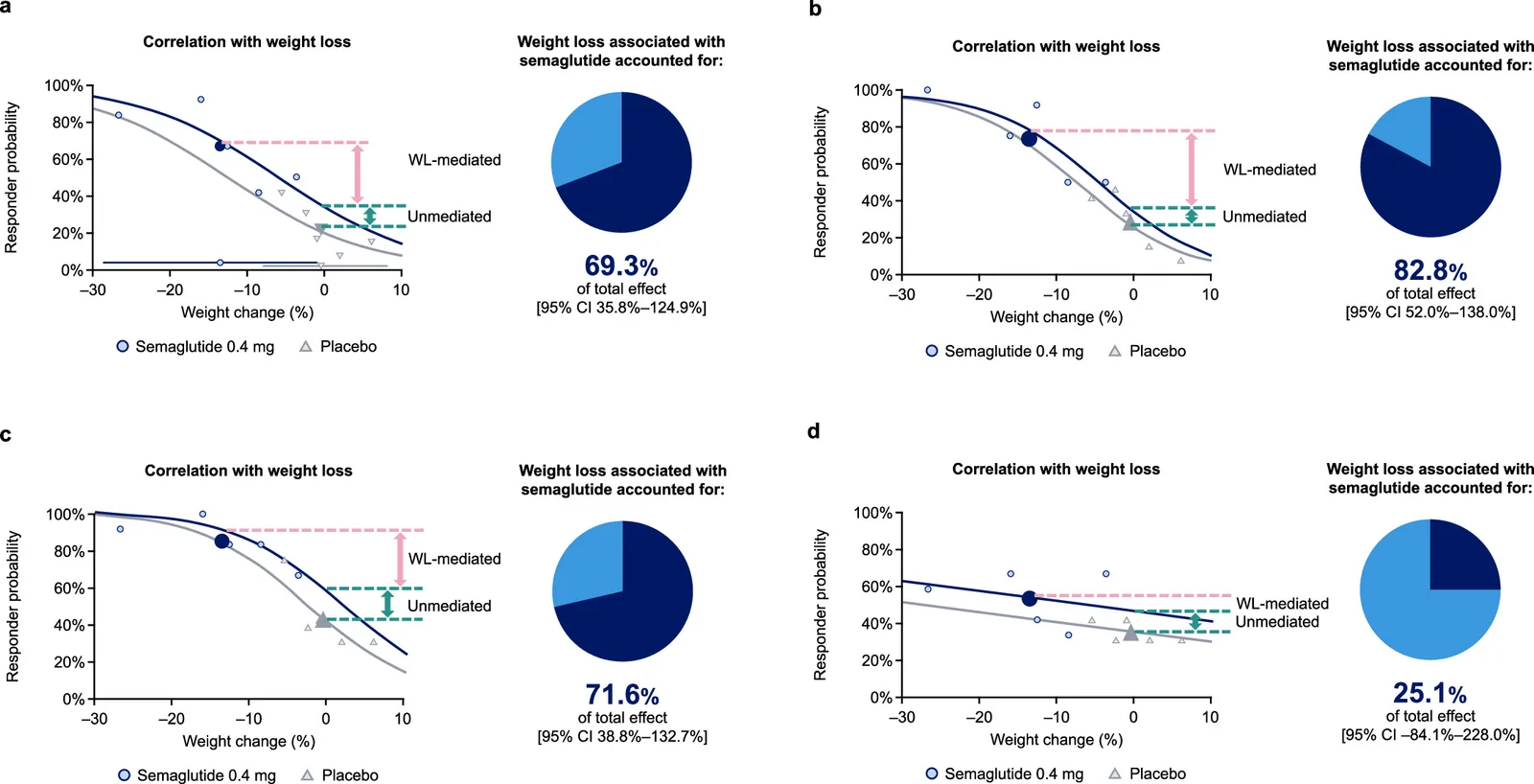
Weight loss effect of semaglutide on MASH resolution (a), steatosis improvement (b), hepatocyte ballooning (c) and liver fibrosis improvement (d). Data based on complete-case on-treatment measurements. Scatter points show responder rates for five weight-loss categories. Enlarged data points show the overall mean body-weight loss versus overall responder rate. Outcome model: logistic regression at week 72. Mediators: weight loss at weeks 4, 12, 20, 28, 36, 44, 52, 62 and 72. Baseline confounders: age, gender, type 2 diabetes status, fibrosis stage and body weight. MASH, metabolic dysfunction-associated steatohepatitis; WL, weight loss. Source data

In a phase 2 trial involving patients with biopsy-confirmed MASH and liver fibrosis stages 1–3, semaglutide 0.4 mg once daily resulted in significantly greater weight loss (13% versus 1%) and higher rates of resolution of steatohepatitis without worsening of fibrosis versus placebo (59% versus 17%) (*P* < 0.001) after 72 weeks of treatment^5^. To investigate whether semaglutide improved liver histology solely through weight loss or via mechanisms that may be separate from weight loss, a mediation analysis using natural effects models was performed. Weight loss directly mediated a substantial proportion of MASH resolution without worsening of fibrosis (69.3% of total effect (95% confidence interval (CI): 35.8–124.9)) (Fig. 1 and Extended Data Fig. 1). Consistent with this, weight loss also directly mediated a major part of the improvement in steatosis (82.8% (95% CI: 52.0–138.0)) and hepatocyte ballooning (71.6% (95% CI: 38.8–132.7)). Conversely, the observed improvement in histologically assessed fibrosis was mediated through weight loss to a lesser extent (25.1% of total effect (95% CI: −84.1 to 228.0)). This finding indicates that, although weight loss is the predominant mediator of effect, factors other than weight loss may also play a role in the histological improvements associated with semaglutide. Using the same patient population from the phase 2 trial^5^, in addition to body weight, semaglutide 0.4 mg was associated with significantly greater improvements in multiple cardiometabolic risk factors versus placebo (Fig. 2a). In all patients evaluated on-treatment at week 72, a descriptive analysis showed that changes in most of the assessed metabolic measures were correlated with achieving resolution of MASH without worsening of fibrosis (Fig. 2b–j).

### Semaglutide effect on markers of hepatic steatosis

We further evaluated the effects of semaglutide on proteomic surrogates of histological components of MASH. Serum samples were collected from phase 2 trial participants after 72 weeks of treatment. A SomaScan aptamer-based proteomics approach was employed using a predefined suite of SomaSignal non-alcoholic steatohepatitis (NASH) tests validated against liver histology to grade/stage steatosis, lobular inflammation, hepatocellular ballooning and liver fibrosis comprising 12, 14, 5 and 8 protein analytes, respectively^7,8^ (Supplementary Table 1).

**Fig. 3:**
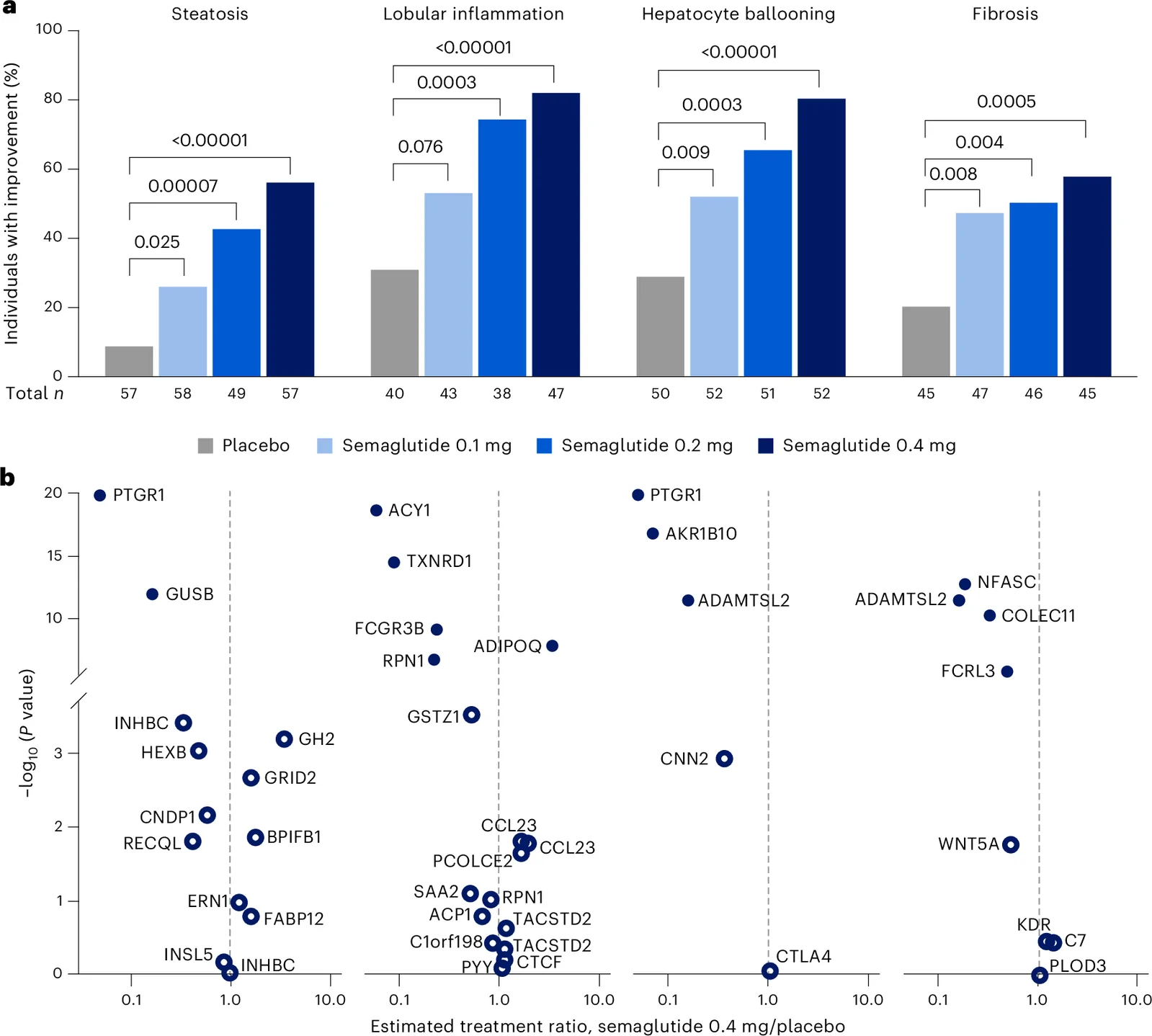
Improvements in MASH with semaglutide assessed by aptamer-based SomaSignal NASH tests. **a**, Proportion of individuals with improvement in each MASH component. Improvement was defined as a negative SomaSignal test at week 72 in individuals with a positive test at baseline. Proportions of individuals with improvement were compared between semaglutide and placebo by a linear-by-linear association test for ordered data. ***P < 0.001. Analysis was two-sided with estimated treatment ratios derived from a multivariable-adjusted MMRM, with no adjustment for multiple testing. **b**, Volcano plots showing the estimated treatment ratio of semaglutide 0.4 mg/placebo and associated *P* value for all individual markers included in each SomaSignal NASH test. For each marker, the effect of semaglutide 0.4 mg versus placebo was tested in an MMRM analysis. Statistically significant treatment ratios of semaglutide 0.4 mg/placebo were evaluated using a two-sided Bonferroni-adjusted family-wise error rate of <0.1. Filled circles denote statistical significance; open circles denote no statistical significance. The presence of duplicate genes in **b** is due to two different targets covering the same gene and protein. ACP1, acid phosphatase 1; ACY1, aminoacylase-1; ADAMTSL2, a disintegrin and metalloproteinase with thrombospondin motifs-like protein 2; ADIPOQ, adiponectin; AKR1B10, aldo-keto reductase family 1 member B10; BPIFB1, bactericidal/permeability-increasing-fold-containing family B member 1; C1orf198, uncharacterized protein C1orf198; C7, complement component C7; CCL23, C-C motif chemokine 23; CNDP1, beta-Ala-His dipeptidase; CNN2, calponin-2; COLEC11, collectin-11; CTCF, transcriptional repressor CTCF; CTLA4, cytotoxic T-lymphocyte protein 4; ERN1, serine/threonine-protein kinase/endoribonuclease IRE1; FABP12, fatty acid-binding protein 12; FCGR3B, low-affinity immunoglobulin gamma Fc region receptor III-B; FCRL3, Fc receptor-like protein 3; GH2, growth hormone 2; GRID2, glutamate receptor ionotropic, δ-2; GSTZ1, maleylacetoacetate isomerase; GUSB, β-glucuronidase; HEXB, β-hexosaminidase B; INHBC, inhibin beta C chain; INSL5, insulin-like peptide 5; KDR, vascular endothelial growth factor receptor 2; NFASC, neurofascin; PCOLCE2, procollagen C-endopeptidase enhancer 2; PLOD3, procollagen-lysine, 2-oxoglutarate 5-dioxygenase 3; PTGR1, prostaglandin reductase 1; PYY, peptide YY; RECQL, ATP-dependent DNA helicase Q1; RPN1, dolichyl-diphosphooligosaccharide–protein glycosyltransferase subunit 1; SAA2, serum amyloid A-2 protein; TACSTD2, tumor-associated calcium signal transducer 2; TXNRD1, thioredoxin reductase 1; WNT5A, protein Wnt-5a. Source data

**Extended Data Fig. 2:**
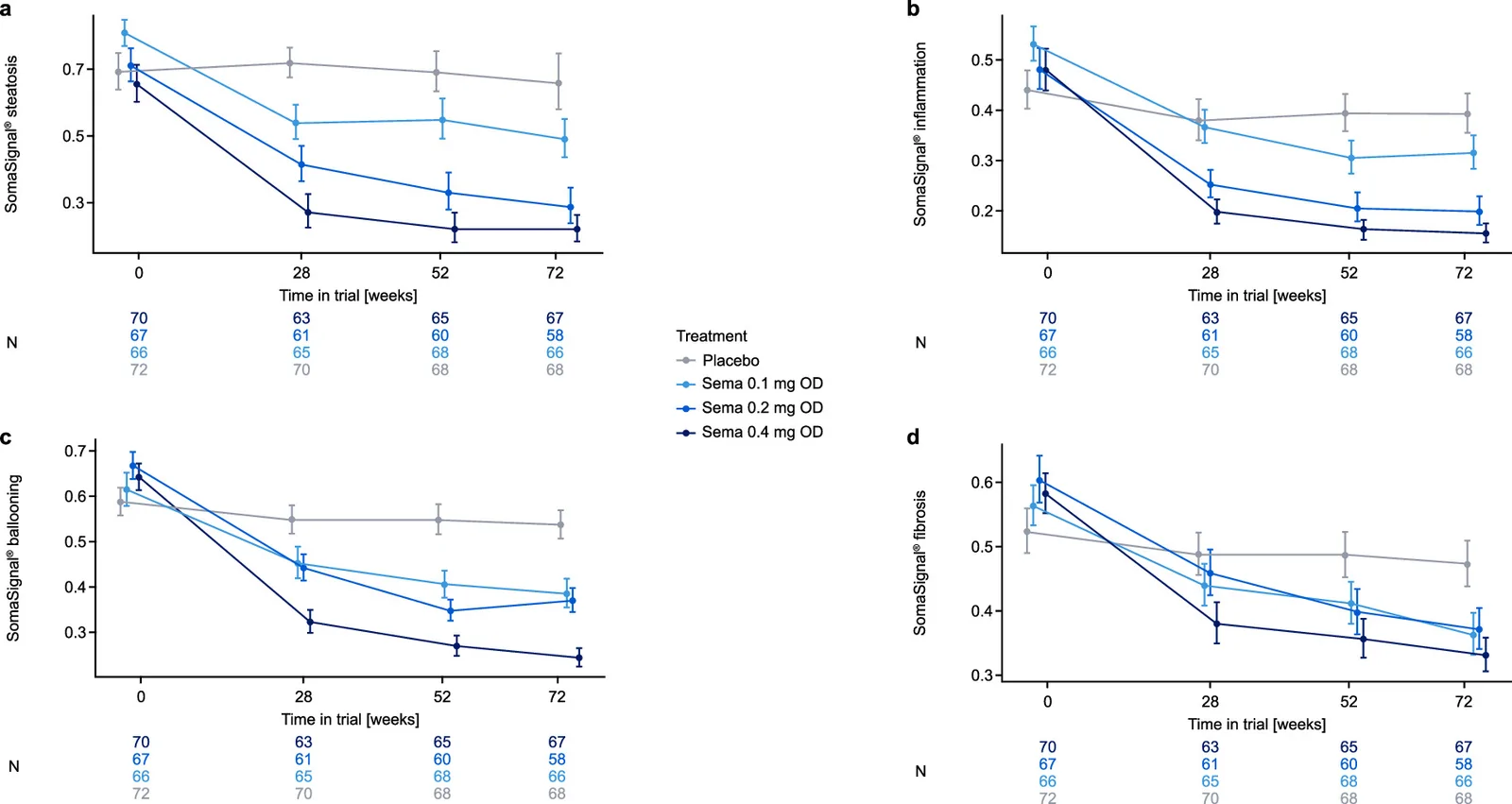
SomaSignal NASH tests for steatosis (a), inflammation (b), ballooning (c) and fibrosis (d) show a dose-dependent response to semaglutide treatment. The full population was analyzed. No technical replicates were used. Error bars are geometric means with standard error of the mean. MASH, metabolic dysfunction-associated steatohepatitis; *N*, number of observations; OD, once daily; Sema, semaglutide. Source data

The SomaSignal test for steatosis showed a dose-dependent response to semaglutide treatment (Fig. 3a and Extended Data Fig. 2). In a subgroup of patients with predicted steatosis score ≥1 at baseline, semaglutide significantly increased the proportion of individuals predicted to have resolution of steatosis (that is, S < 1) after 72 weeks of treatment. For all semaglutide treatment arms, a statistically significant proportion of individuals achieved an improvement in SomaSignal-defined steatosis after 72 weeks versus placebo (semaglutide 0.1 mg 26%, 0.2 mg 43%, 0.4 mg 55% and placebo 9%) (Fig. 3a). Applying a sensitivity analysis, estimated treatment ratios for individual protein analytes were then determined. Of 12 proteins in the SomaSignal test for steatosis, two (PTGR1 and GUSB) showed a statistically significant lower abundance for semaglutide 0.4 mg versus placebo (Fig. 3b).

### Semaglutide effect on hepatic inflammation and ballooning

The SomaSignal tests for inflammation and ballooning showed a dose-dependent response to semaglutide treatment (Fig. 3a and Extended Data Fig. 2). In a subgroup of patients with SomaSignal-predicted inflammation stage ≥2 at baseline, semaglutide significantly increased the proportion of participants estimated to have inflammation stage <2 after 72 weeks of treatment, with a clear dose–response relationship (semaglutide 0.1 mg 53%, 0.2 mg 71%, 0.4 mg 82% and placebo 32%). Similar findings were observed for SomaSignal-defined hepatocyte ballooning (semaglutide 0.1 mg 52%, 0.2 mg 65%, 0.4 mg 80% and placebo 29%) (Fig. 3a). A significant estimated treatment ratio for semaglutide 0.4 mg versus placebo was observed for three (PTGR1, AKR1B10 and ADAMTSL2) out of five protein analytes for hepatocyte ballooning and five (ACY1, TXNRD1, FCGR3B, ADIPOQ and RPN1) out of 14 proteins for SomaSignal-defined lobular inflammation (Fig. 3b).

### Semaglutide effect on markers of hepatic fibrosis

It is important to recognize that fibrosis stage is the product of an altered equilibrium between fibrogenesis and fibrolysis. Although the phase 2 clinical trial did not demonstrate significance over placebo in achieving regulatory-defined histological improvement in fibrosis stage at 72 weeks, this regulatory endpoint is based solely on histologically demonstrating increased regression but does not capture the clinical and biological relevance of reduced progression of fibrosis. Dose-dependent reductions in liver stiffness assessed by FibroScan and enhanced liver fibrosis test score were observed with semaglutide, as were significant reductions in disease progression^5^. In the present study, the SomaSignal test for fibrosis showed a dose-dependent response to semaglutide treatment (Fig. 3a and Extended Data Fig. 2). In a subgroup with predicted fibrosis stage ≥2 at baseline, semaglutide significantly increased the proportion of individuals with predicted fibrosis <2 after 72 weeks of treatment with a clear dose–response relationship. Consistently, the number of participants with a normalization of SomaSignal-defined fibrosis increased across all treatment arms with a clear dose–response relationship (semaglutide 0.1 mg 44%, 0.2 mg 48%, 0.4 mg 57% and placebo 16%) (Fig. 3a). In a sensitivity analysis, four out of 11 protein analytes were significantly different in the semaglutide 0.4 mg group versus placebo: ADAMTSL2, NFASC, COLEC11 and FCRL3 (Fig. 3b).

**Extended Data Fig. 3:**
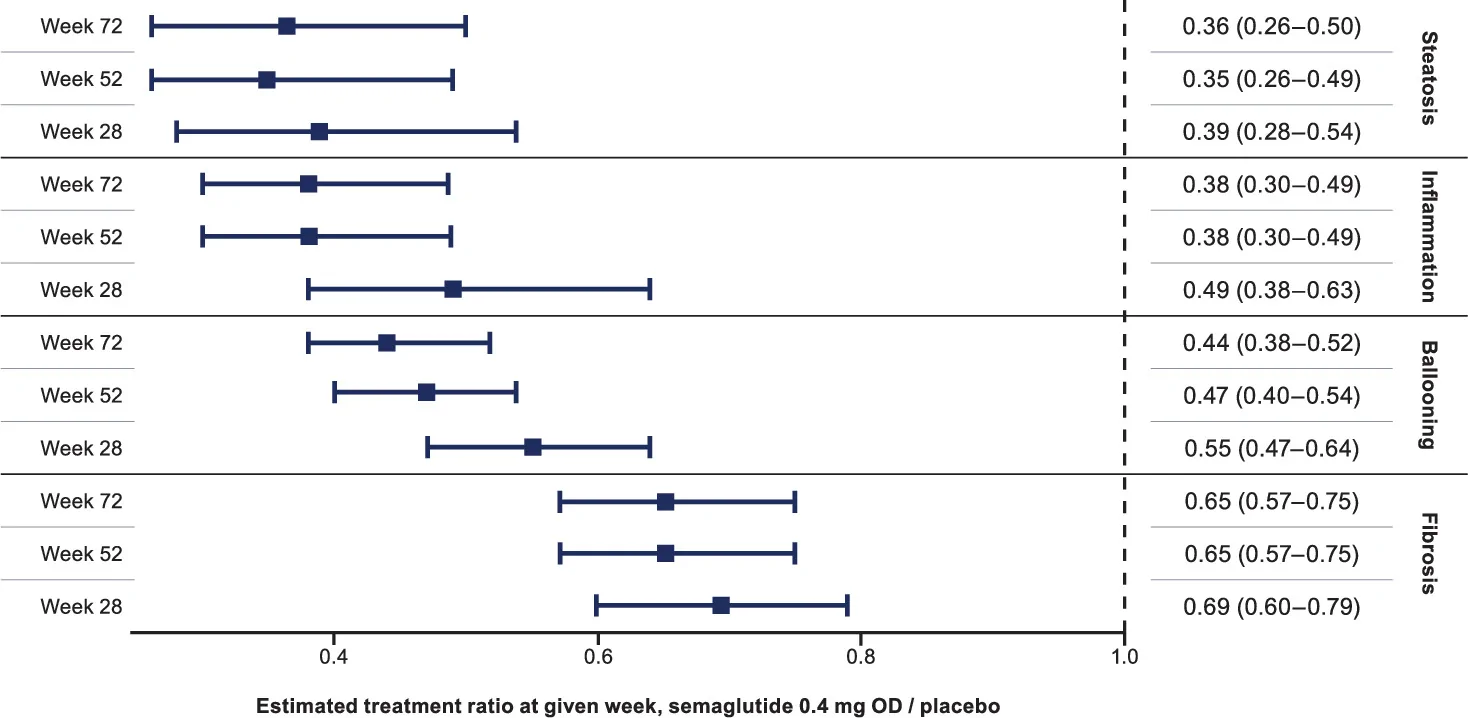
Treatment effects in the semaglutide 0.4 mg group versus the placebo group at weeks 28, 52 and 72 for each of the four SomaSignal NASH tests. Mixed model for repeated measurements. Data are presented as estimated treatment ratio and 95% confidence intervals. For each component of MASH, the number of patients at week 28, 52 and 72 was 63, 65 and 67, respectively. MASH, metabolic dysfunction-associated steatohepatitis; OD, once daily. Source data

Overall, the greatest treatment effect was seen for SomaSignal-defined steatosis, followed by inflammation, ballooning and fibrosis (estimated greater reductions with semaglutide 0.4 mg versus placebo at week 72 of 64%, 62%, 56% and 35%, respectively) (Extended Data Fig. 3).

### Semaglutide effects on liver fibrosis: preclinical models

**Extended Data Fig. 4:**
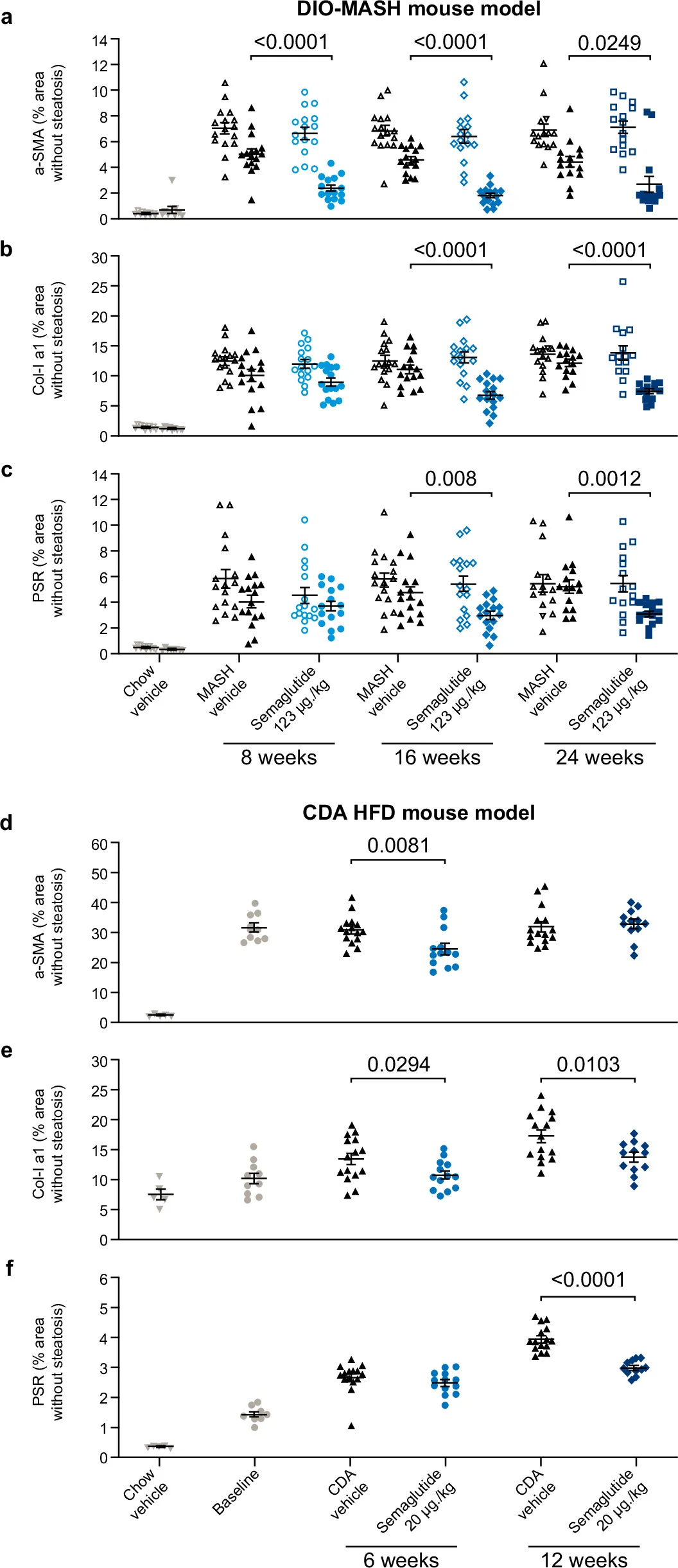
Semaglutide treatment in DIO-MASH and CDA-HFD mice: effects of treatment duration on liver fibrosis. Effects of once-daily subcutaneous semaglutide on fibrogenesis/fibrosis markers αSMA (**a, d**), Col-I α1 (**b, e**) and PSR (**c, f**) in DIO-MASH mice (**a-c**) and CDA-HFD mice (**d-f**). DIO-MASH: Open symbols show pretreatment values. Chow vehicle (grey triangles) for 24 weeks (*n* = 9), MASH vehicle and semaglutide 123 µg/kg for 8 weeks (*n* = 16 [black triangles] and *n* = 16 [light blue circles], respectively), 16 weeks (*n* = 16 [black triangles] and *n* = 16 [blue rectangles]), and 24 weeks (*n* = 15 [black triangles] and *n* = 15 [dark blue square]). Differences between semaglutide and DIO-MASH vehicle were assessed using Welch’s unpaired t-test, two tailed. CDA-HFD: Chow vehicle for 12 weeks (*n* = 5 [grey triangles]), baseline prior to treatment (*n* = 10 [grey circles]), CDA-HFD vehicle or semaglutide 20 µg/kg for 6 weeks (*n* = 15 [black triangles] and *n* = 13 [blue circles], respectively) and 12 weeks (*n* = 15 [black triangles] and *n* = 12 [dark blue triangles], respectively). Differences between semaglutide and CDA-HFD vehicle were assessed using Welch’s unpaired t-test, two tailed. Data are presented as mean values ± s.e.m. αSMA, αsmooth muscle actin; CDA-HFD, choline-deficient L-amino acid-defined high-fat diet; Col-I α1, α-1 type I collagen; DIO, diet-induced obesity; MASH, metabolic dysfunction-associated steatohepatitis; PSR, Picrosirius Red; s.e.m., standard error of the mean. Source data

To gain deeper insights into the antifibrotic effects of semaglutide, we used two established mouse models of MASH: diet-induced obesity MASH (DIO-MASH) mice^9^ and choline-deficient L-amino acid-defined high-fat diet (CDA-HFD) mice^10,11^. Like humans, DIO-MASH mice exhibit metabolic features of MASH although with less pronounced fibrosis. CDA-HFD mice represent a non-metabolic (that is, non-obese) model of rapidly progressive steatohepatitis and liver fibrosis. In DIO-MASH mice, there was widespread fibrosis at treatment onset, and fibrosis was significantly reduced compared to pretreatment biopsies and versus vehicle-treated animals after 16 weeks or 24 weeks of semaglutide treatment (Extended Data Fig. 4). In CDA-HFD mice, all mice were lean throughout the study but exhibited persistent liver steatosis. Fibrosis progressed during the treatment phase, and semaglutide significantly improved fibrosis versus vehicle-treated animals. The improvement in fibrosis with semaglutide was slightly less pronounced versus that seen in DIO-MASH mice, whereas early markers of fibrosis showed a sustained effect over the treatment period (Picrosirius Red (PSR) improved over time; type 1 collagen (Col1) was equally significant at two timepoints; and α-smooth muscle actin (αSMA) lost effect over time) (Extended Data Fig. 4).

**Extended Data Fig. 5:**
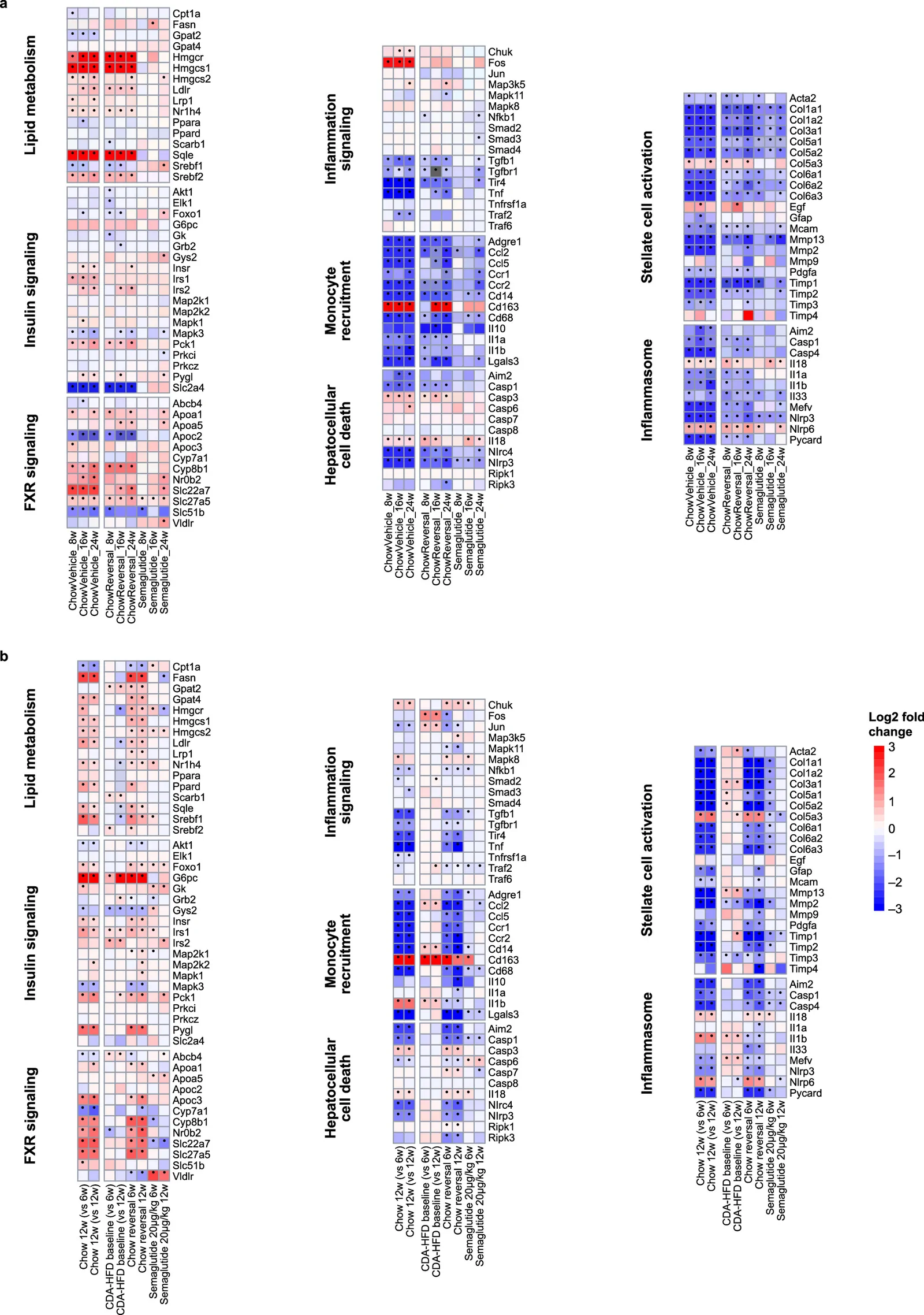
Heatmap depicting relative levels (log fold change) of differentially expressed selected candidate genes associated with MASH and fibrosis in semaglutide-treated DIO-MASH mice (a) and CDA-HFD mice (b). The liver transcriptome was probed against a selected set of genes involved in lipid metabolism, insulin signaling, FXR signaling, inflammation signaling, monocyte recruitment, hepatocellular cell death and stellate cell activation. Upregulated (red color gradient) and downregulated (blue color gradient) gene expression in individual pathways as compared to the corresponding control group. *P < 0.05 versus DIO-MASH vehicle (**a**) and CDA-HFD vehicle (**b**). *P* values corrected for multiple testing using the Benjamini and Hochberg method. CDA-HFD, choline-deficient L-amino acid-defined high-fat diet; DIO, diet-induced obesity; FXR, farnesoid X receptor; MASH, metabolic dysfunction-associated steatohepatitis; W, week.

We probed the liver transcriptome of DIO-MASH and CDA-HFD mice against a predefined set of genes relevant for MASH. In DIO-MASH mice, semaglutide decreased expression of inflammation markers and induced a sustained downregulation of fibrosis-related collagens as well as modulators of fibrosis, such as Timp-1, Timp-2 and MMP13, supporting reduced fibrogenesis and increased fibrolysis in groups treated with semaglutide versus vehicle (Extended Data Fig. 5a). In CDA-HFD mice, semaglutide significantly reduced expression of genes involved in collagen turnover (extracellular matrix remodeling) and pro-inflammatory activity (inflammation signaling, monocyte recruitment and inflammasome). A negligible effect was observed on lipid metabolic genes (lipid metabolism and insulin signaling) (Extended Data Fig. 5b).

### Semaglutide effect on cellular components of the liver

**Extended Data Fig. 6:**
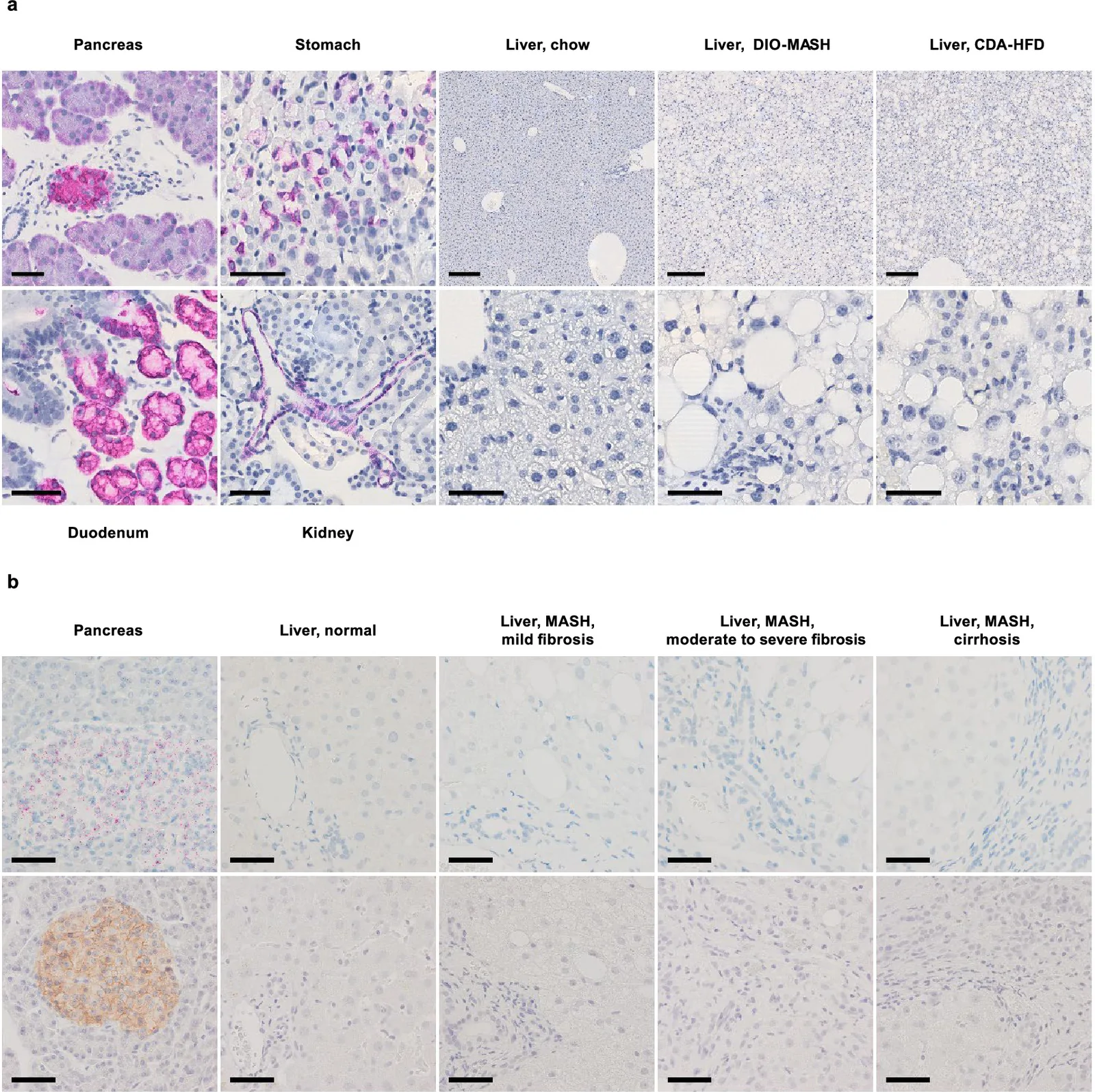
No evidence of GLP-1 receptor expression in mouse and human liver. **a**, Assessment of GLP-1 receptor protein expression in mouse tissue samples by immunohistochemistry. Representative photomicrographs of sections of positive control tissues from C57Bl/6 normal mouse (pancreas, stomach, duodenum and kidney) all showed immunoreactive cell populations. Samples of normal liver (chow; n = 1) and livers from DIO-MASH (n = 4) and CDA-HFD mice (n = 4) are all devoid of GLP-1 receptor immunoreactivity. Scale bars, 50 µm (control tissues and bottom images of liver) or 250 µm (top images of liver). **b**, Assessment of GLP-1 receptor mRNA and protein expression in human liver by RNAscope in situ hybridization (top row) and immunohistochemistry (bottom row) on human tissue samples. Representative photomicrographs of sections of normal-range liver biopsies (n = 6) and liver biopsies from individuals with MASH and mild fibrosis (n = 7), moderate to severe fibrosis (n = 6) or cirrhosis (n = 7). Normal-range human pancreas sample served as a positive control for GLP-1 receptor expression (left). Scale bars, 50 µm. CDA-HFD, choline-deficient L-amino acid-defined high-fat diet; DIO, diet-induced obesity; GLP-1, glucagon-like peptide-1; MASH, metabolic dysfunction-associated steatohepatitis. Source data

Considering the effects of semaglutide on liver pathology observed in humans and mice, we assessed whether semaglutide could potentially directly affect cells within the hepatic microenvironment using RNA in situ hybridization and immunohistochemistry. Neither of these assays detected any GLP-1 receptor mRNA or protein expression in human or mouse liver tissue samples. This included hepatocytes, hepatic stellate cells, Kupffer cells, cholangiocytes and endothelial cells, along with immune cells and macrophages found in the liver tissue (Extended Data Fig. 6).

### Semaglutide effect on circulating protein expression

**Fig. 4:**
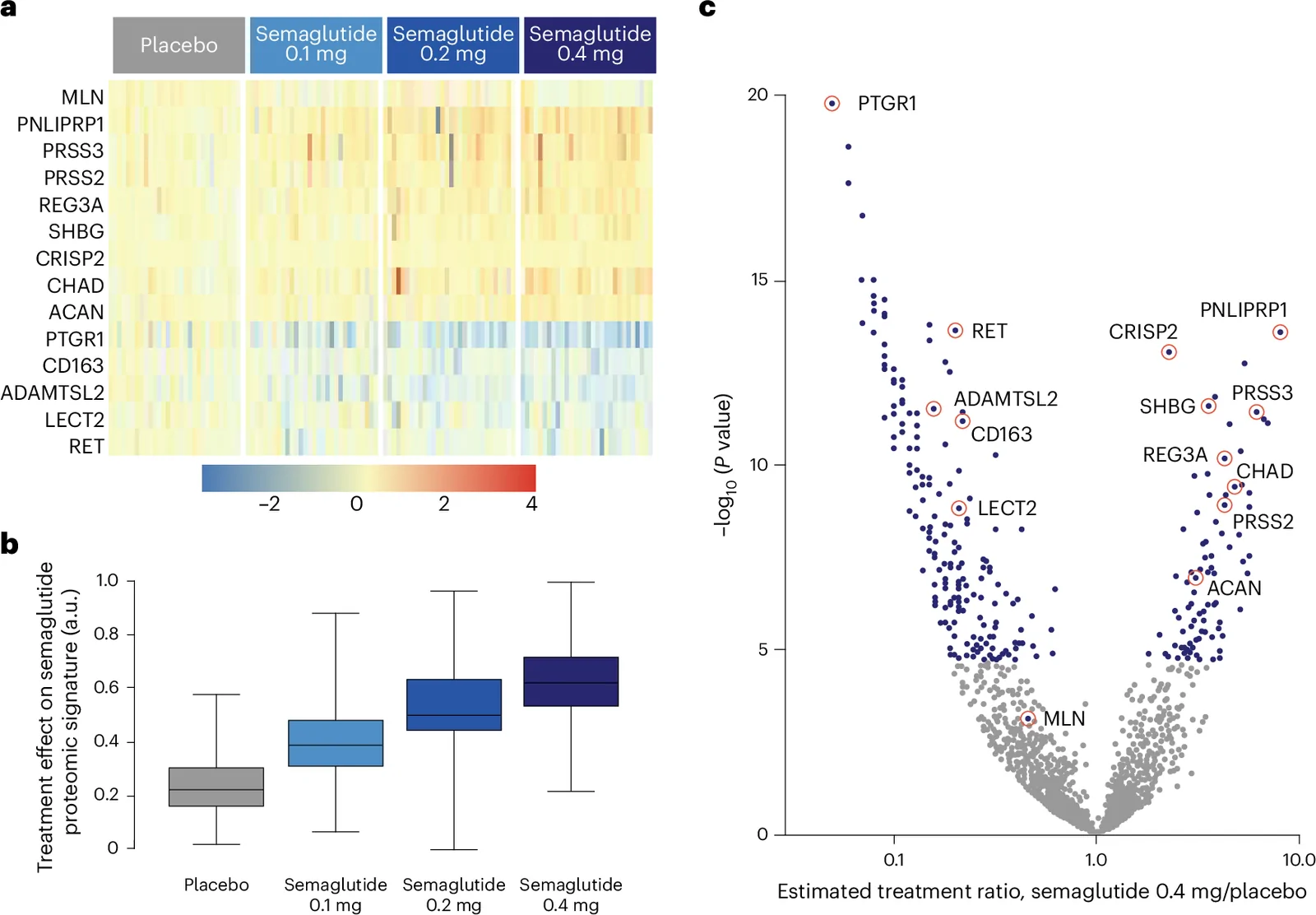
Semaglutide treatment effect on biomarkers included in the aptamer-based SomaScan assay. **a**, Heatmap showing the 14 markers found to constitute the aptamer-based proteomic signature of semaglutide treatment. Data mining was performed using the repeated LASSO procedure including all 4,979 markers in the SomaScan assay as input. For each marker, change from baseline at week 72 was used as predictor variable. The heatmap shows individual changes in protein expression from baseline to week 72 on an arbitrary scale. **b**, Box plot showing the treatment effect of placebo and semaglutide 0.1 mg, 0.2 mg and 0.4 mg on the 14-marker proteomic signature of semaglutide. The treatment effect was calculated from the average LASSO coefficients for each of the 14 markers and is presented on an arbitrary scale. The number of patients is the on-treatment population for each treatment group. a.u., arbitrary units. **c**, Volcano plot showing the estimated treatment ratio of semaglutide 0.4 mg/placebo at week 72 and associated *P* value for all 4,979 individual markers included in the SomaScan assay. For each marker, the effect of semaglutide 0.4 mg versus placebo was tested in an MMRM analysis. Statistically significant treatment ratios of semaglutide 0.4 mg/placebo were evaluated using a two-sided Bonferroni-adjusted family-wise error rate of <0.1. Blue dots denote statistical significance; gray dots denote no statistical significance. Red circles show the 14 markers included in the proteomic signature of semaglutide treatment (see **b**). ACAN, aggrecan core protein; ADAMTSL2, a disintegrin and metalloproteinase with thrombospondin motifs-like protein 2; CD163, scavenger receptor cysteine-rich type 1 protein M130; CHAD, chondroadherin; CRISP2, cysteine-rich secretory protein 2; LECT2, leukocyte cell-derived chemotaxin-2; MLN, promotilin; PNLIPRP1, inactive pancreatic lipase-related protein 1; PRSS2, trypsin-2; PRSS3, trypsin-3; PTGR1, prostaglandin reductase 1; REG3A, regenerating islet-derived protein 3 alpha; RET, (REarranged during Transfection) receptor tyrosine kinase; SHBG, sex hormone-binding globulin. Source data

Leveraging the full potential of the SomaScan serum proteomics platform, we performed data mining to identify key proteins associated with semaglutide treatment exposure in the phase 2 trial. Applying the least absolute shrinkage and selection operator (LASSO) procedure including all 4,979 proteins in the SomaScan assay as input, a group of semaglutide-responsive proteins emerged, consisting of a subset of 14 proteins that best profiled the changes in the semaglutide 0.4 mg group (Fig. 4a). To obtain information on the signature’s fit in all trial participants, we calculated the treatment effect from the average LASSO coefficient for each of the 14 identified proteins. The resulting models followed a clear dose response, showing least treatment effect on the signature in the placebo group and the highest in the semaglutide 0.4 mg group (Fig. 4b).

To determine the effect of semaglutide 0.4 mg versus placebo on all SomaScan proteins, we tested for each of the 4,979 markers in a mixed model and obtained significance for 279 proteins, which included 13 of the previously identified 14 semaglutide-responsive proteins (Fig. 4c). We next aimed to determine which biological processes are represented by those proteins that significantly changed with semaglutide treatment. We used the well-annotated hallmark gene sets from the Molecular Signatures Database^12^. A gene set enrichment analysis was performed, and model estimates for the treatment ratio of semaglutide 0.4 mg versus placebo at week 72 for all 4,979 proteins in the SomaScan assay were included. Effects on proteins with annotation in 14 of the 50 hallmark gene sets were seen with semaglutide treatment, most of which can be linked to relevant biological pathways and processes in the context of MASH (Extended Data Table 1).

### Semaglutide effect on proteins associated with MASH resolution

We next sought to identify proteins that were statistical mediators of the effect of semaglutide on MASH resolution. The analysis was based on the presence of MASH resolution irrespective of treatment arm, with baseline weight included as a confounding variable in the main mediation model. We identified a ‘treatment signature’ comprising 72 unique proteins that was significantly associated with semaglutide 0.4 mg and MASH resolution (Table 1). With the exception of FCGR3B, ADIPOQ and RPN1 for hepatic inflammation and ballooning, and FCRL3 for fibrosis, the treatment signature included all of the proteins in the SomaSignal MASH tests that were significantly different in the semaglutide 0.4 mg group versus placebo. We found 45 of the 72 semaglutide signature proteins to be represented in the Molecular Signatures Database hallmark gene sets. Given the finding that weight loss accounted for most of the beneficial effect of semaglutide in MASH, we ascertained whether any of the 72 proteins remained significantly associated with semaglutide-induced MASH resolution after accounting for weight loss. A linear regression model that included weight change plus the full list of confounders was fitted in the placebo arm comparing change in proteins versus weight loss. For 26 proteins, no association was observed between weight loss and protein change in placebo patients. Thus, these 26 proteins may reflect semaglutide-induced MASH resolution that could be, at least in part, separate from weight loss (Table 1); however, further investigation and independent validation are required.

**Table 1:** Proteins from the SomaScan assay that are significantly associated with the semaglutide effect on MASH resolution

SomaScan ID	Full name	Entrez gene ID	Entrez gene name	Hallmark	Treatment ratio^a^	FDR-adjusted *P* value^b^	Separate from weight loss in the present study^c^	Proteins significantly associated with MASLD diagnosis^13^	Proteins significantly associated with cirrhosis^13^	Proteins significant for fibrosis stage F3–4 vs baseline F0–2 identified in LITMUS cohort^14^	Proteins significant for NAS ≥ 4 vs NAS < 4 identified in LITMUS cohort^14^
11265-8	Retinal dehydrogenase 1	216	ALDH1A1	Bile acid metabolism Fatty acid metabolism Peroxisome	0.083	0.008	Y	Y	Y		Y
13967-14	Thioredoxin reductase 1, cytoplasmic	7296	TXNRD1	Mtorc1 signaling ROS pathway	0.094	0.008	Y	Y			Y
15487-164	Liver carboxylesterase 1	1066	CES1	Xenobiotic (drug) metabolism	0.114	0.008		Y		Y	
15525-294	Alcohol dehydrogenase 1C	126	ADH1C	Fatty acid metabolism Xenobiotic (drug) metabolism	0.075	0.008	Y	Y	Y		Y
15534-26	Malate dehydrogenase, mitochondrial	4191	MDH2	Adipogenesis Fatty acid metabolism Glycolysis Oxidative phosphorylation	0.186	0.008					
15562-24	Beta-glucuronidase	2990	GUSB	Cholesterol homeostasis Glycolysis	0.151	0.008		Y	Y		Y
17755-5	UDP-glucose 6-dehydrogenase	7358	UGDH	Estrogen response late Fatty acid metabolism Xenobiotic (drug) metabolism	0.063	0.008	Y	Y	Y		Y
18173-11	Aflatoxin B1 aldehyde reductase member 3	22977	AKR7A3	No hallmark annotation	0.114	0.008	Y	Y	Y	Y	Y
18185-118	Fructose-bisphosphate aldolase B	229	ALDOB	Glycolysis Hypoxia	0.073	0.008	Y	Y	Y	Y	Y
18206-18	Alcohol dehydrogenase 6	130	ADH6	No hallmark annotation	0.092	0.008	Y	Y	Y		
19262-219	Very long-chain specific acyl-CoA dehydrogenase, mitochondrial	37	ACADVL	Fatty acid metabolism Oxidative phosphorylation	0.189	0.008		Y			
19617-5	Prostaglandin reductase 1	22949	PTGR1	Xenobiotic (drug) metabolism	0.055	0.008	Y	Y	Y	Y	Y
3344-60	Antithrombin-III	462	SERPINC1	Coagulation Complement IL-2/STAT5 signaling	5.423	0.008	Y				
6379-62	ADAMTS-like protein 2	9719	ADAMTSL2	No hallmark annotation	0.158	0.008	Y	Y	Y	Y	Y
9213-24	Formimidoyltransferase-cyclodeaminase	10841	FTCD	Heme metabolism	0.085	0.008	Y	Y	Y	Y	Y
17758-79	L-xylulose reductase	51181	DCXR	Estrogen response late P53 pathway Xenobiotic (drug) metabolism	0.101	0.010	Y	Y	Y	Y	Y
3343-1	Aminoacylase-1	95	ACY1	No hallmark annotation	0.058	0.013	Y	Y	Y	Y	Y
6049-64	Receptor-type tyrosine-protein phosphatase S	5802	PTPRS	No hallmark annotation	5.549	0.018		Y	Y		
11441-11	Glycogen phosphorylase, liver form	5836	PYGL	Glycolysis	0.105	0.019	Y	Y			
17675-17	Acyl-coenzyme A thioesterase 13	55856	ACOT13	No hallmark annotation	0.222	0.019					
2625-53	Hsp90alpha	3320	HSP90AA1	Fatty acid metabolism	0.095	0.019	Y	Y			
17808-37	Omega-amidase NIT2	56954	NIT2	No hallmark annotation	0.183	0.020		Y			
18397-5	Aldo-keto reductase family 1 member C4	1109	AKR1C4	No hallmark annotation	0.095	0.020	Y	Y	Y	Y	Y
12370-30	Apolipoprotein F	319	APOF	No hallmark annotation	7.046	0.020		Y	Y	Y	Y
18188-12	Glycine amidinotransferase, mitochondrial	2628	GATM	No hallmark annotation	0.114	0.020	Y	Y	Y	Y	
3503-4	Integrin alpha-I: beta-1 complex	3672/3688	ITGB1/ITGA1	No hallmark annotation	0.105	0.020		Y	Y	Y	Y
9832-33	Homogentisate 1,2-dioxygenase	3081	HGD	No hallmark annotation	0.079	0.020	Y	Y	Y	Y	Y
11241-8	Argininosuccinate lyase	435	ASL	Xenobiotic (drug) metabolism	0.085	0.021		Y	Y	Y	Y
12334-25	Serine hydroxymethyltransferase, cytosolic	6470	SHMT1	E2F targets	0.155	0.021		Y			
17150-8	10-kDa heat shock protein, mitochondrial	3336	HSPE1	Mtorc1 signaling MYC targets V1 MYC targets V2	0.166	0.021		Y	Y		
17341-89	Acetyl-CoA acetyltransferase, cytosolic	39	ACAT2	Cholesterol homeostasis Fatty acid metabolism	0.122	0.021	Y	Y			
17396-23	Alcohol dehydrogenase 1A	124	ADH1A	No hallmark annotation	0.089	0.021		Y	Y	Y	Y
17783-9	Cob(I)yrinic acid a,c-diamide adenosyltransferase, mitochondrial	326625	MMAB	No hallmark annotation	0.294	0.021		Y			
18186-15	Threonine-tRNA ligase, cytoplasmic	6897	TARS1	Unfolded protein response UV response (up)	0.182	0.021			Y		
2731-29	NADPH-cytochrome P450 reductase	5447	POR	Adipogenesis Oxidative phosphorylation Xenobiotic (drug) metabolism	0.127	0.021	Y	Y	Y	Y	Y
3009-3	Transforming growth factor beta receptor type 3	7049	TGFBR3	Apoptosis Epithelial-to-mesenchymal transition UV response (down)	3.181	0.021		Y			
7206-20	Fructose-1,6-bisphosphatase 1	2203	FBP1	Hypoxia Xenobiotic (drug) metabolism	0.088	0.021		Y	Y	Y	Y
18342-2	Phosphoserine aminotransferase	29968	PSAT1	Mtorc1 signaling Unfolded protein response	0.072	0.023		Y	Y		Y
19197-95	Acetyl-CoA acetyltransferase, mitochondrial	38	ACAT1	Oxidative phosphorylation	0.155	0.024		Y	Y	Y	
16081-38	Aldo-keto reductase family 1 member B10	57016	AKR1B10	KRAS signaling (down)	0.077	0.024		Y	Y	Y	Y
17787-1	Enoyl-CoA hydratase, mitochondrial	1892	ECHS1	Adipogenesis Fatty acid metabolism Oxidative phosphorylation	0.134	0.024		Y	Y	Y	
13954-9	Glucosamine 6-phosphate N-acetyltransferase	64841	GNPNAT1	No hallmark annotation	0.115	0.025	Y	Y			
17748-21	Quinone oxidoreductase PIG3	9540	TP53I3	No hallmark annotation	0.151	0.026		Y			
15447-45	Sorbitol dehydrogenase	6652	SORD	Androgen response Estrogen response late Mtorc1 signaling MYC targets V2	0.080	0.026		Y	Y		Y
16307-22	Netrin receptor UNC5D	137970	UNC5D	No hallmark annotation	4.998	0.026	Y	Y			
18338-26	Isocitrate dehydrogenase (NADP) cytoplasmic	3417	IDH1	Adipogenesis Bile acid metabolism Fatty acid metabolism Glycolysis Mtorc1 signaling Oxidative phosphorylation Peroxisome Xenobiotic (drug) metabolism	0.105	0.026		Y			
11424-4	Fumarylacetoacetase	2184	FAH	Adipogenesis IL-2/STAT5 signaling Xenobiotic (drug) metabolism	0.092	0.029		Y	Y		Y
15308-108	Brorin	375567	VWC2	No hallmark annotation	2.510	0.030					
13460-4	Chondroadherin	1101	CHAD	No hallmark annotation	4.129	0.030	Y	Y	Y		
17377-1	Aldo-keto reductase family 1 member C3	8644	AKR1C3	Xenobiotic (drug) metabolism	0.195	0.034	Y	Y	Y		Y
5467-15	Heat shock protein HSP 90-beta	3326	HSP90AB1	MYC targets V1	0.097	0.034	Y	Y			
17138-8	Glutathione S-transferase A1	2938	GSTA1	No hallmark annotation	0.088	0.034		Y	Y		Y
11257-1	Dihydropteridine reductase	5860	QDPR	Adipogenesis Mtorc1 signaling	0.085	0.035		Y			
17712-7	Isopentenyl-diphosphate delta-isomerase 1	3422	IDI1	Androgen response Bile acid metabolism Cholesterol homeostasis Fatty acid metabolism Mtorc1 signaling Peroxisome	0.162	0.035		Y			
7179-69	Neurofascin	23114	NFASC	Apical junction	0.181	0.035		Y	Y	Y	
9359-9	Protein delta homolog 2	65989	DLK2	KRAS signaling (down)	2.883	0.035		Y			
18398-1	3-oxo-5-beta-steroid 4-dehydrogenase	6718	AKR1D1	Bile acid metabolism	0.117	0.037		Y	Y		Y
8325-37	Alcohol dehydrogenase 4	127	ADH4	Cholesterol homeostasis	0.088	0.039		Y	Y		Y
15481-45	Antibacterial protein LL-37	820	CAMP	No hallmark annotation	2.031	0.041					
9805-51	Semaphorin-4B	10509	SEMA4B	No hallmark annotation	2.113	0.041					
8229-1	Glucoside xylosyltransferase 1	283464	GXYLT1	No hallmark annotation	1.855	0.042		Y			
9876-20	Fructose-bisphosphate aldolase C	230	ALDOC	Cholesterol homeostasis Hypoxia	0.121	0.042		Y	Y		
11368-32	Adenylate kinase 2, mitochondrial	204	AK2	Adipogenesis E2F targets	0.135	0.043					
13983-27	Quinone oxidoreductase	1429	CRYZ	Fatty acid metabolism	0.180	0.043		Y	Y	Y	Y
4430-44	Collectin-11	78989	COLEC11	No hallmark annotation	0.297	0.043		Y	Y	Y	Y
13580-2	UDP-N-acetylhexosamine pyrophosphorylase	6675	UAP1	Androgen response	0.454	0.045		Y	Y		
5463-22	Growth arrest-specific protein 1	2619	GAS1	Apical surface Epithelial-to-mesenchymal transition	2.668	0.045			Y		
15499-11	Attractin	8455	ATRN	UV response (down)	0.400	0.047		Y	Y		
4962-52	Cerebral dopamine neurotrophic factor	441549	CDNF	No hallmark annotation	1.802	0.047			Y		
6392-7	WNT1-inducible-signaling pathway protein 2	8839	CCN5	Estrogen response early Estrogen response late Hypoxia	3.054	0.047					
6627-25	Inactive pancreatic lipase-related protein 1	5407	PNLIPRP1	No hallmark annotation	8.679	0.047	Y	Y	Y		
18882-7	Calsyntenin-2	64084	CLSTN2	No hallmark annotation	0.519	0.048		Y	Y	Y	

A classical mediation analysis was performed to identify proteomic changes that mediate the effect of semaglutide on MASH resolution. All proteins were used as input. The method by Dai et al.^37^ was used to adjust for multiple testing and compute FDR-adjusted mediation *P* values. For 72 proteins, the mediation *P* value was less than 0.05.

^a^Semaglutide 0.4 mg versus placebo.

^b^*q* value.

^c^Proteins identified as significantly associated with semaglutide-induced MASH resolution after accounting for weight loss.

F, fibrosis; IL-2, interleukin 2; KRAS, Kirsten rat sarcoma virus; ROS, reactive oxygen species; STAT5, signal transducer and activator of transcription 5; Y, yes.

Source data

### Semaglutide reverses the MASH proteomic signature

To investigate whether MASH affects the abundance of the 72 proteins identified in the treatment signature, we investigated serum levels from a subset of participants in the Copenhagen Cohort of MASLD (CoCoMASLD, formerly known as the FLINC (Fatty Liver Disease in Nordic Countries) cohort; ClinicalTrials.gov: NCT04340817, H-17029039) whose datasets included clinical measurements and archived samples. SomaScan data analysis of 235 participants was available: 146 with MASH and 89 healthy volunteers.

**Extended Data Fig. 7:**
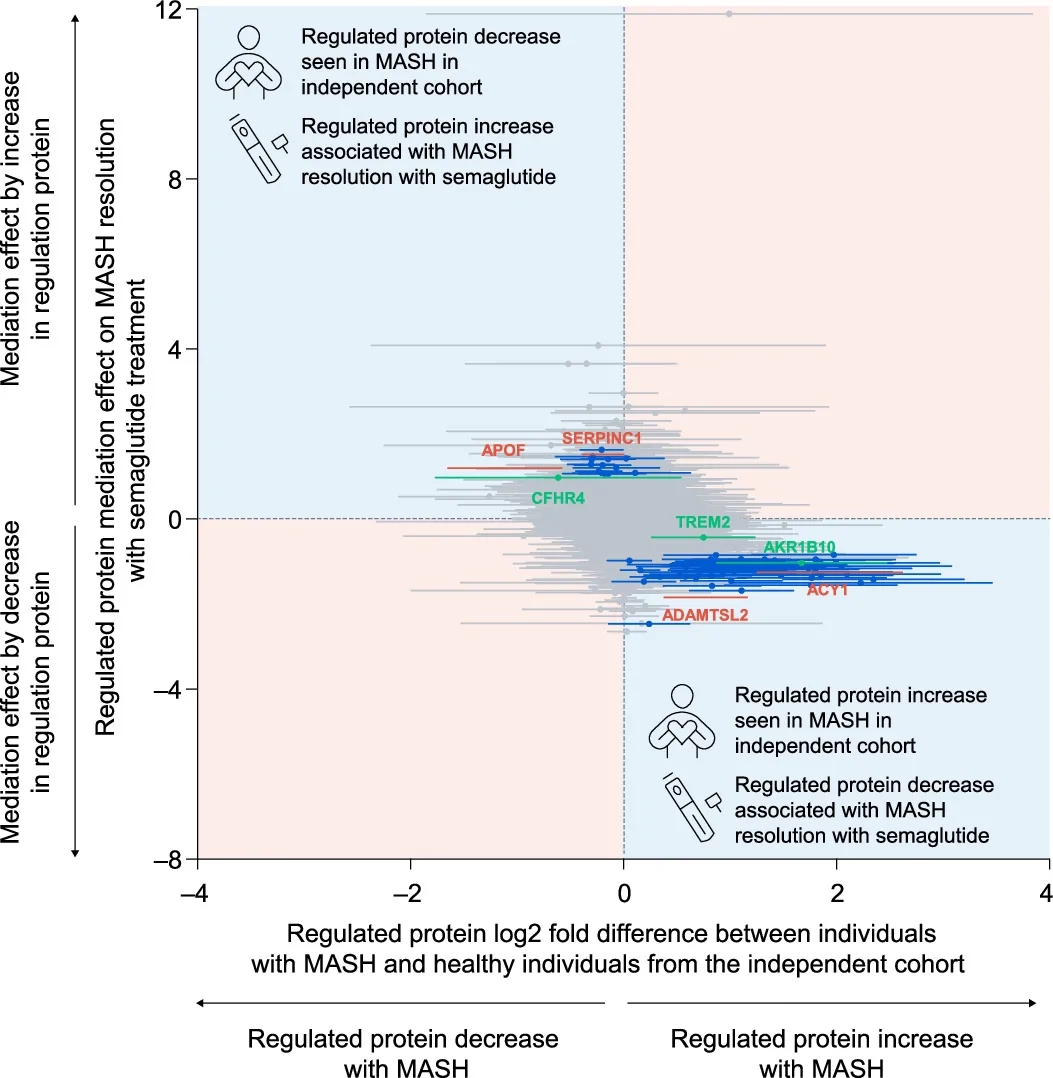
Change in protein levels in participants with MASH treated with semaglutide compared with protein levels in healthy volunteers in an independent real-world observational cohort. Gray-colored data points and error bars represent the full complement of aptamer-based SomaScan proteins; blue-colored points and error bars are those proteins significantly associated with semaglutide-induced MASH resolution; proteins of interest in the current work are highlighted in orange and green. The vertical axis represents the mediation effect on protein biomarkers from the SomaScan analysis with respect to semaglutide-induced MASH resolution. The horizontal axis displays the log2 fold changes in protein levels between healthy individuals and those with MASH in the independent CoCoMASLD cohort, while accounting for the effect of gender, age, body mass index and diabetes status. Source data

Analysis confirmed that the same 72 proteins identified in semaglutide-treated patients with MASH were also differentially expressed in patients with MASH relative to healthy individuals in the independent cohort (Extended Data Fig. 7). By way of example, we present data for several proteins of interest based on the following rationale: SERPINC1 and APOF had among the highest treatment ratios in the present work (Table 1). In previous proteomic analyses, ACY1 had the strongest association for MASLD compared to population controls^13^, and analysis of the LITMUS Metacohort identified ADAMTSL2, AKR1B10, CFHR4 and TREM2 as significantly associated with MASH and clinically significant fibrosis^14^.

**Fig. 5:**
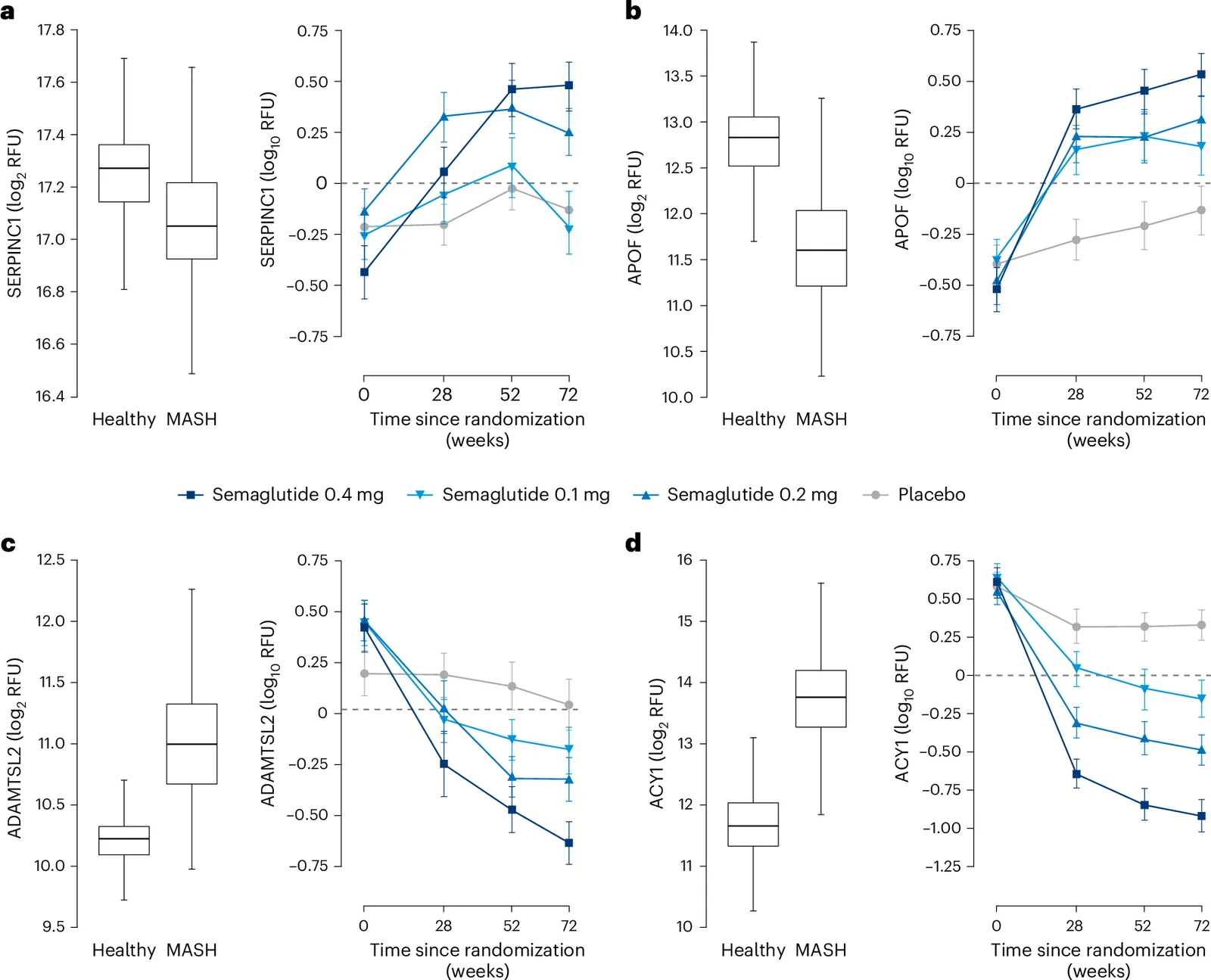
Change in protein levels of participants with MASH treated with semaglutide compared to protein levels in healthy volunteers in an independent real-world observational cohort. **a**. Left-hand panel shows abundance of SERPINC1 from healthy individuals and those with MASH in the independent cohort. Center of box plot is median; box boundary is first and third quantiles; upper whisker is third quantile plus 1.5 IQR; and lower whisker is first quantile minus 1.5 IQR, where IQR is the third quantile minus the first quantile (healthy, *n* = 89; MASH, *n* = 146). The right-hand panel shows the effect of semaglutide treatment on SERPINC1 levels in patients with MASH from a phase 2 trial population (semaglutide 0.1 mg (*n* = 80), semaglutide 0.2 mg (*n* = 78), semaglutide 0.4 mg (*n* = 82) and placebo (n = 80)). **b**–**d**, As for **a** but showing levels of APOF, ADAMTS2 and ACY1, respectively. No technical replicates were used. Data are presented as mean + s.e.m. ADAMTSL2, a disintegrin and metalloproteinase with thrombospondin motifs-like protein 2; APOF, apolipoprotein F; ACY1, aminoacylase-1; IQR, interquartile range; SERPINC1, serpin family C member 1. s.e.m., standard error of the mean. Source data

**Extended Data Fig. 8:**
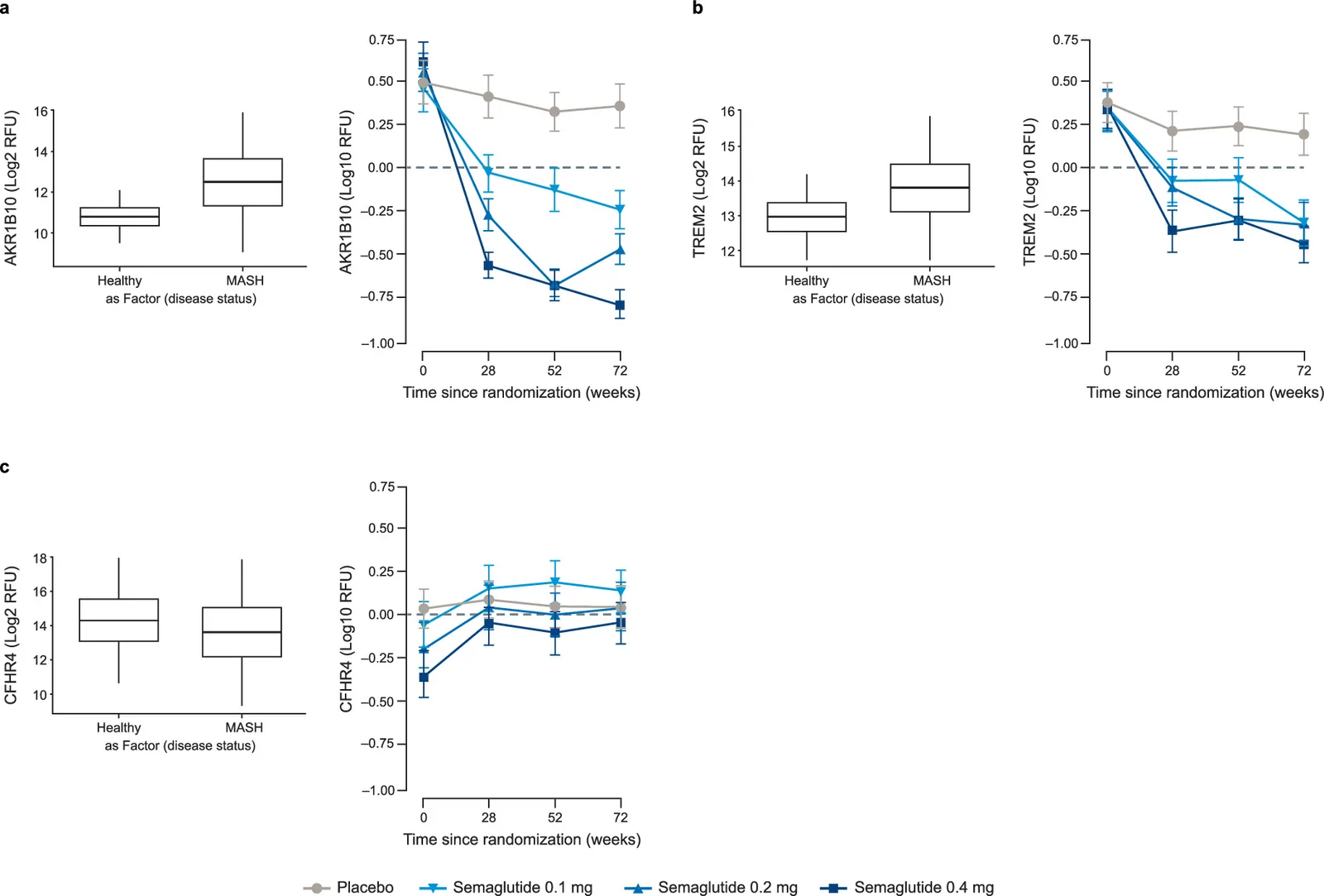
Change in AKR1B10, TREM2 and CFHR4 protein levels in participants with MASH treated with semaglutide compared with protein levels in healthy volunteers in an independent real-world observational cohort. **a**. Left-hand panel shows abundance of AKR1B10 from healthy individuals and those with MASH in the independent cohort. Center of boxplot is median, box boundary is first and third quantile, upper whisker is third quantile plus 1.5 IQR and lower whisker is first quantile minus 1.5 IQR, where IQR is third quantile minus first quantile (healthy, n = 89; MASH, n = 146). The right-hand panel shows the effect of semaglutide treatment on AKR1B10 levels in patients with MASH from a phase 2 trial population (semaglutide 0.1 mg [n = 80], semaglutide 0.2 mg [n = 78], semaglutide 0.4 mg [n = 82] and placebo [n = 80]). **b** and **c**, as for panel **a** but showing levels of TREM2 and CFHR2, respectively. Data are presented as mean + s.e.m. MASH, metabolic dysfunction-associated steatohepatitis; RFU, relative fluorescence units; s.e.m., standard error of the mean. Source data

Levels of SERPINC1 and APOF in the independent cohort were reduced in patients with MASH versus healthy individuals (Fig. 5a,b, left panel). In patients from the phase 2 trial, semaglutide treatment increased the levels of both proteins from baseline to week 72 (Fig. 5a,b, right panel). In contrast to SERPINC1 and APOF, levels of ADAMTSL2 and ACY1 were elevated in patients with MASH versus healthy individuals from the independent cohort (Fig. 5c,d, left panel) but decreased after semaglutide treatment (Fig. 5c,d, right panel). The same pattern was observed for AKR1B10, CFHR4 and TREM2 identified in the LITMUS Metacohort^14^ (a lesser effect for CFHR4 was attributed to minimal differences between healthy individuals and those with MASH and the relatively high baseline value for placebo) (Extended Data Fig. 8). Comparing treatment signature changes in semaglutide-treated patients with MASH to protein expression levels in patients with MASH relative to healthy individuals in the independent cohort, semaglutide appeared to reverse the altered levels of the 72 proteins in MASH to the pattern observed in healthy individuals (Extended Data Fig. 7 and Table 1).

## Discussion

Achieving resolution of MASH implies that any potential treatment should have a meaningful impact on inflammation, cellular damage and fibrogenesis^15^. Here we show that semaglutide achieved significant dose-dependent improvements in aptamer-based SomaSignal-defined tests for steatosis, inflammation, ballooning and fibrosis that correlate with histological components of MASH^7^. A deeper SomaScan analysis identified 72 unique proteins that were significantly associated with MASH resolution and semaglutide treatment. Notably, a treatment-associated proteomic signature in MASH has not previously been reported. Notably, too, the treatment signature comprises proteins previously identified as being associated with chronic liver disease. With the exception of 11 proteins in the semaglutide treatment signature, the remaining 61 proteins were identified as significantly associated with MASLD diagnosis compared to population controls in a proteomic analysis by Sveinbjornsson et al.^13^ (Table 1). Additionally, two-thirds of the proteins in the semaglutide treatment signature were found to be associated with cirrhosis (for example, fibrotic burden) versus population controls^13^. In a cross-sectional proteo-transcriptomic analysis reported by the LITMUS consortium, most of the proteins identified as being significant for MASH versus non-MASH were also found in the semaglutide treatment signature and, to a lesser extent, also for F3–F4 versus F0–F2 (ref. ^14^) (Table 1). The inverse correlation in most of the 72 proteins identified in an independent real-world cohort versus the phase 2b trial cohort suggests that semaglutide may reverse the MASH proteomic phenotype to one similar to healthy individuals. This parallels the preclinical work of Rakipovski et al.^16^ who reported that semaglutide significantly reversed expression of genes in pathways relevant to the pathogenesis of atherosclerosis in aortic tissue.

The mechanistic basis of semaglutide-induced MASH resolution has been variably attributed to effects on weight and inflammation as well as reductions in metabolic dysfunction and lipotoxic effects^16–18^. The present work supports these findings but also raises the possibility that additional mechanisms may contribute to the overall therapeutic effect. Mediation analysis suggested that 26 of the 72 proteins in the treatment signature may contribute to semaglutide-induced MASH resolution in a manner that may not be fully explained by weight loss. It is acknowledged, however, that more detailed interrogation of these specific proteins is required to fully establish any effect separate from weight loss. Given the lack of GLP-1 receptor expression on hepatocytes^19^, which is corroborated in the present work in human and mouse liver tissue samples, the beneficial effects observed with semaglutide support an extrahepatic mechanism of action or an intermediate signaling effect from the periphery to the liver. Studies in mice have highlighted potential for a small involvement of GLP-1 receptors in intrahepatic T cells, endothelial cells and circulating monocytes; however, the precise location and functional importance of these cell types for the beneficial actions are unknown^20,21^.

The weight loss effects of semaglutide are well described in humans and mechanistically informative animal studies and involve broadly expressed brain GLP-1 receptors that mediate effects to increase satiety, reduce hunger and change food choice and preference^22^. The effects that reduce inflammation are less well understood, but some T cells, and Brunner’s glands in the intestine, express GLP-1 receptors^16,20,23^. Moreover, two independent populations of neurons in the dorsal vagal complex and nucleus tractus solitarius are important for the control of peripheral inflammation, and both express GLP-1 receptors that are targeted by semaglutide^24,25^. In the present work, semaglutide reduced expression of inflammation markers and genes involved in pro-inflammatory activity in mouse models of MASH. As noted above, SomaSignal NASH tests for lobular inflammation and hepatocyte ballooning demonstrated a significant dose-dependent effect of semaglutide in samples from humans. This finding is supported in the phase 2b trial in which a numerically greater improvement in lobular inflammation and hepatocyte ballooning was observed in participants treated with semaglutide versus placebo.

Inflammation is a major driver of liver fibrosis, and the primary risk of MASH is progressive fibrosis leading to cirrhosis. In the present work, SomaSignal NASH tests demonstrated improvement in predicted liver fibrosis. Although weight loss accounted for a major part of histological improvement of MASH with semaglutide, particularly for improvements in disease activity, observed improvement in fibrosis was mediated by weight loss to a lesser extent. In both DIO-MASH and CDA-HFD mice, we found significant changes in markers of early and late fibrosis as well as extracellular accumulation of collagen. The effect of semaglutide in the DIO-MASH model is tightly associated with weight loss and reduced liver steatosis and demonstrates the potential for GLP-1RA treatment to improve both metabolic and histologic aspects of MASH, including fibrosis regression linked to body weight. In the non-obese CDA-HFD model, fibrosis improvement obtained after prolonged treatment is indicative of disease-modifying mechanisms largely unrelated to body weight and systemic metabolic alteration. This finding is noteworthy given that an antifibrotic effect is observed in a mouse model with no or minimal systemic metabolic disease despite the lack of GLP-1 receptors in the liver. Liver transcriptome analysis further revealed downregulation of fibrosis-related genes with semaglutide treatment in both mouse models. Taken together, these observations support an antifibrotic effect of semaglutide via modification of systemic biological processes. Notably, in ESSENCE, a significantly higher proportion of patients receiving semaglutide 2.4 mg showed an improvement in liver fibrosis without any deterioration in steatohepatitis versus placebo (36.8% versus 22.4%; *P* < 0.001), clarifying the effect of semaglutide on liver fibrosis^6^.

Patients with MASH have cardiometabolic risk factors and are at high risk for liver fibrosis and atherosclerotic cardiovascular disease^26^. Thus, therapeutic strategies to prevent liver fibrosis and atherosclerotic cardiovascular disease are required for the treatment of MASH^26^. Preclinical^16^ and clinical evidence have demonstrated a cardiovascular protective effect of semaglutide in individuals with diabetes^27^ and separately in individuals with obesity^28^. Within this context, the present work identified ADAMTSL2 and ACY1 as two aptamer-based protein mediators significantly associated with semaglutide-induced MASH resolution that are implicated in cardiovascular disease and fibrosis. ADAMTSL2 regulates the extracellular microenvironment^29^, is associated with myocardial fibrosis^30^ and adverse outcomes in patients with heart failure^31^ and was shown to be a biomarker to identify significant and advanced fibrosis in patients with MASLD in the LITMUS Metacohort^14^. ACY1 is associated with myocardial fibrosis in mouse heart failure^32^, is overexpressed in liver tissue from humans with obesity and MASLD^14^, has the strongest association for MASLD diagnosis versus population controls^13^ and may be a biomarker for predicting future development of T2D^33^. Thus, ACY1 appears to play an interconnected role in metabolic diseases that are risk factors for cardiovascular disease and MASH.

APOF and SERPINC1 are implicated in lipid metabolism and tumorigenesis, respectively. APOF controls plasma and hepatic lipoprotein metabolism^34^ and has been identified as a potential biomarker for progression of non-alcoholic fatty liver disease^14^. APOF is selectively expressed in the liver and may be a candidate biomarker for liver status in MASH. SERPINC1 acts as a suppressor of HCC^35^. Given the increased risk of HCC in individuals with MASH, there may be downstream clinical implications associated with semaglutide-induced modulations in HCC-specific pro-carcinogenic genes. Although clinical evidence is limited, GLP-1RAs were associated with a lower risk of first-time diagnosis of HCC versus non-GLP-1RA glucose-lowering medications in patients with T2D^36^. Future work is warranted to examine long-term associations of semaglutide with HCC incidence in patients with T2D and obesity.

Our work has several strengths. The proteomic analysis was performed at multiple timepoints on biosamples from participants in a randomized controlled trial in MASH, the results of which are reflected in real-world proteomic data. The identified treatment signature has a high degree of overlap not only with SomaSignal MASH tests validated against biopsy results but also with proteomic analyses from other large-scale population-based studies in MASLD and MASH, suggesting that the findings are generalizable. Additionally, animal data were derived from proven preclinical models of MASH.

Our work also has limitations. Although preclinical data have provided some insights into potential mechanisms, we acknowledge that neither of the mouse models fully replicates the histopathological features of human MASH^11^. Our clinical results are based on samples derived from participants in a phase 2b trial. It is not possible, at this time, to validate our findings in the phase 3 ESSENCE trial population given the ongoing nature of the trial and the availability of only interim data on histological endpoints. However, evaluating a correlation of proteomic signature to clinical endpoints may be feasible once the ESSENCE trial is complete. Although we report changes in abundance of proteins significantly associated with semaglutide-induced MASH resolution, the aptamer-based proteomics assay is semiquantitative, and so absolute concentrations of proteins are not evaluated. Despite applying a widely accepted statistical approach to evaluate the effect of body weight on treatment response, the analysis reports semaglutide-induced weight loss only (that is, weight loss is a proxy for the changes in body composition that are causally linked to treatment response). It remains to be seen whether findings can be transferred to other interventions that result in a similar reduction of body weight through a different change in body composition. However, with the effect of semaglutide-induced weight loss on histological outcome (that is, the indirect/mediated effects) being lower than the effect of randomizing participants to semaglutide or placebo on the histology outcomes (that is, the total effects), we think that this provides a rationale for the existence of additional factors beyond weight loss, but further investigations are required to test this hypothesis. Although every participant received nutritional and physical activity counseling, we did not collect information on dietary factors or physical activity. Lastly, improved diet and exercise might have improved histology without weight change.

In summary, our data suggest that semaglutide may achieve resolution of MASH predominantly via weight loss but also through modulation of metabolic and inflammatory pathways that exert indirect effects on hepatic tissue, which include mechanisms of fibrogenesis, with some of the explored protein aptamers implicated in cardiovascular disease and cancer risk. The ability of semaglutide to modulate or reverse multiple pathogenic pathways in complex disease may be one of its defining characteristic features.

## Methods

### Phase 2b clinical trial

The data and analyses derive from a 72-week, multicenter, randomized, parallel-group trial of semaglutide versus placebo, the results of which were published in detail previously^5^. In brief, this trial involved 320 adult participants (18–75 years of age (20–75 years of age in Japan)), with or without T2D and a body mass index higher than 25 kg m^−2^ at screening with histological evidence of MASH (defined as the presence of at least grade 1 steatosis, lobular inflammation and hepatocyte ballooning with an overall non-alcoholic fatty liver disease activity score (NAS) of 4 or higher) and a fibrosis stage of F1, F2 or F3 based on the Kleiner fibrosis classification. NAS and the fibrosis stage were assessed centrally by two independent expert hepatologists who were blinded to treatment assignment, patient characteristics and each other’s assessment. In case of different opinions on any variable, diagnostic agreement was reached through a consensus call.

Liver-related exclusion criteria were documented causes of chronic liver disease other than MASH, in particular hepatitis B (including positive hepatitis B surface antigen), hepatitis C (including positive HCV-RNA) and alcoholic liver disease or known or suspected abuse of alcohol (>20 g per day for women or >30 g per day for men), alcohol or narcotics dependence assessed by the Alcohol Use Disorders Identification Test (AUDIT questionnaire), liver transplantation, elevated liver tests (liver enzymes >5 times the upper normal limit, elevated total bilirubin (>1.5 mg dl^−1^) and international normalized ratio >1.3) and treatment with vitamin E or pioglitazone that has not been at a stable dose in the period for 90 days prior to screening or from historical baseline biopsy until screening, respectively. Glucose-related and obesity-related exclusion criteria included glycated hemoglobin (HbA1c) > 10% at screening, treatment with GLP-1RAs or sodium/glucose co-transporter 2 inhibitors in the period from 90 days prior to screening or from historical baseline biopsy until screening, treatment with any other glucose-lowering agent not stable in the period from 28 days prior to screening or from historical baseline liver biopsy until screening, participation in an organized weight reduction program, previous surgical treatment for obesity and any treatment with a medication that could promote weight loss.

Participants, stratified by geographic region, T2D status and fibrosis stage, were randomized to receive semaglutide at a maximum dose of 0.1 mg (*n* = 80), 0.2 mg (*n* = 78) or 0.4 mg (*n* = 82) or placebo (*n* = 80) via once-weekly subcutaneous injection for 72 weeks. Biopsy samples obtained at screening were used as baseline for histologic variables, and an additional biopsy was obtained at week 72. In addition to per-protocol laboratory analyses, human biosamples for future analysis were retained, as long as participants had signed a separate informed consent form. Stored fasting serum from participants was used to perform large-scale proteomic profiling using the SomaScan multiplex affinity assay.

This trial accorded with the ethical principles of the Declaration of Helsinki and was consistent with the International Conference on Harmonization of Good Clinical Practice and applicable regulatory requirements. The protocol was approved by the institutional review board and ethics committee at each participating trial site^5^. Participants provided written informed consent for use of biosamples for future research, and the ethics committees approved the specific use of the biosamples.

In total, 94% (*n* = 302) of participants completed the trial (that is, accomplished the final trial visit), and 89% (*n* = 285) completed treatment. In 87% (*n* = 277) of randomized participants, liver biopsy at week 72 was available to evaluate the primary endpoint of resolution of MASH and the confirmatory secondary endpoint of improvement of at least one fibrosis stage and no worsening of MASH. Outcomes were calculated with missing values imputed as non-responders.

Among participants included in this trial, 61% (*n* = 193) were women, 78% (*n* = 248) were White and 13% (*n* = 40) were Hispanic or Latino. In total, 51% (*n* = 163) of trial participants were enrolled in Europe and Australia, 36% (*n* = 116) in North America and 13% (*n* = 41) in Japan.

Baseline demographics and disease characteristics were generally similar across treatment groups. Mean participant age was 55 years; mean body weight was 98.4 kg; and mean body mass index was 35.8 kg m^−2^. One hundred sixty-five out of 320 randomized patients (52%) had a body mass index of 35 kg m^−2^ or higher.

### Mediation analysis of the contribution of weight loss to MASH improvement

To investigate whether semaglutide improved liver histology through weight loss or via mechanisms separate from weight, mediation analyses using natural effects models were performed. The analyses were based on complete-case on-treatment measurements (*N* = 249) for histological parameters that showed a statistically significant effect of semaglutide. Changes from baseline in weight at all nine scheduled visits were used as a mediator. The model was adjusted for baseline body weight, fibrosis stage, T2D status, age and gender. The analyses assessed the mediated (weight-loss-dependent) and non-mediated (after adjusting for weight change—that is, separate from weight loss) effects of treatment on liver histology. Results are presented as the mediated proportion—that is, the indirect/weight-loss-dependent effect divided by the total effect, with corresponding CIs. Covariates were imputed using single imputation based on predicted value/response from a mixed model for repeated measurements (MMRM). The mediation analysis was performed using the ‘medflex’ package in R.

### Correlation between MASH resolution and improvement in features of the metabolic syndrome

Changes in features of the metabolic syndrome at week 72 are presented by treatment and MASH resolution response at week 72 using descriptive statistics based on the on-treatment observation period. To investigate the efficacy of semaglutide on the metabolic and inflammatory features, post-baseline changes were analyzed using an MMRM, with baseline diabetes status, baseline fibrosis stage and diabetes-by-fibrosis interaction as factors (HbA1c, fasting plasma glucose, homeostatic model assessment of insulin resistance, adipose tissue insulin resistance and non-high-density lipoprotein cholesterol could not be included in the diabetes-by-fibrosis interaction as factors) and baseline body weight and baseline value of the feature as covariates, all nested within visit. All scheduled post-baseline assessments during the on-treatment period were used in the model.

### SomaScan proteomic profiling and SomaSignal NASH tests in participants with biopsy-confirmed MASH

Serum samples for future use were obtained at four timepoints in the phase 2 trial: at baseline and after 28, 52 and 72 weeks of treatment. For some participants, samples were not shipped to SomaLogic for analysis due to lack of consent or availability of samples in the storage facility upon the cutoff date for the use of samples. After preprocessing and quality control of the SomaLogic data, a total of 10 serum samples failed. Thus, 1,088 serum samples were included in the SomaLogic analyses, representing 293 of the enrolled 320 participants.

#### SomaScan assay

The SomaScan protein array v.4 profiled 4,979 different proteins. Standard preprocessing of the SomaScan array data was performed in accordance with guidance from SomaLogic. The relative fluorescence units (RFU) from each sample were normalized based on (1) hybridization controls on each microarray to correct for systematic variability in hybridization and (2) median signal based on all features for each dilution to correct for variability across plates according to the SomaScan Data Standardization guidelines (SomaScan Data Standardization and File Specification Technical Note (SSM-020)). All RFUs were natural logarithm transformed for uniformity.

#### SomaSignal NASH tests

The protein expression levels measured using the SomaScan protein array were used for the multi-protein SomaSignal NASH tests. The SomaSignal NASH tests provide categorizations of participants for each of the four MASH components: steatosis, hepatocyte ballooning, lobular inflammation and fibrosis. An overview of the individual proteins from the SomaScan protein array that are included in each of the four SomaSignal NASH tests is presented in Supplementary Table 1. SomaSignal NASH tests used predicted probabilities (continuous score from 0 to 1), with the higher the predicted probability, the higher the likelihood of elevated NAS component and fibrosis stage. Predicted probability was used to categorize the test as positive or negative based on a decision threshold of 0.5 (validated for identification of stage: steatosis ≥1, inflammation ≥2, ballooning ≥1 and fibrosis ≥2). In a subgroup analysis, changes in predicted probabilities were analyzed by an MMRM based on on-treatment data. The proportion of patients with improvement was defined as negative SomaSignal NASH test (predicted probability <0.5 on a 0–1 scale) at week 72.

#### Individual SomaScan markers changing with semaglutide treatment

An MMRM was used with treatment, baseline diabetes status, baseline fibrosis stage and diabetes-by-fibrosis interaction as factors and baseline body weight and baseline value of the biomarker as covariates, all nested within visit. Statistical significance was based on a Bonferroni-adjusted alpha level of 0.1 for the treatment ratio at week 72 of semaglutide 0.4 mg once daily/placebo.

#### Semaglutide proteomic signature

A protein signature was developed for the semaglutide treatment effect (0.4 mg versus placebo; response variable in model). Data mining using the (repeated) LASSO procedure was done using all biomarkers as input. For each marker, change from baseline at week 72 was used (predictor variables). The average area under the concentration time curve for classifying semaglutide 0.4 mg versus placebo from internal cross-validation was 0.92.

#### Effect of semaglutide on Hallmark gene sets

A gene set enrichment analysis was performed. As input, model estimates were used for the treatment ratio at week 72, semaglutide 0.4 mg/placebo, for all 4,979 protein biomarkers.

#### Biomarkers mediating the semaglutide effect on MASH resolution

To identify protein biomarkers that statistically mediate the effect of semaglutide on MASH resolution, two models were fitted for all 4,979 proteins. First, we used a linear regression model of semaglutide treatment versus protein change at week 72. Second, we used a logistic regression model of protein change versus MASH resolution, both at week 72. The same covariates were included as in the MMRM. *P* values from both models were combined, following the classical framework for causal mediation, to compute a false discovery rate (FDR)-adjusted mediation *P* value following the procedure by Dai et al.^37^.

#### Protein mediators of the semaglutide effect on MASH resolution separate of weight loss

We used two procedures to identify whether the protein mediators for the effect of semaglutide on MASH resolution are separate from change in body weight. First, a linear regression was fitted for the association of protein change versus change in body weight, both at week 72. The same covariates were included as in the MMRM. We used a *P* > 0.05 threshold to indicate independence. Second, we repeated the above procedure using two models that included baseline body weight and weight change at week 72 as covariates in the model. Markers that had *P* < 0.05 for both models were considered to mediate separate from body weight.

### Change in proteomic mediators in participants with biopsy-confirmed MASH in the independent cohort

The independent CoCoMASLD cohort comprised patients with MASLD referred to a single gastroenterology department in Denmark. The patient subset included the initially recruited patients who underwent a biopsy and were included in proteomic analyses as well as healthy controls. Patients were diagnosed as having MASH based on histopathological evaluation. The pathologists provided NASH Clinical Research Network scores for steatosis and fibrosis but only yes/no for lobular inflammation and ballooning. Hence, the patients could not be assigned a NAS. Patients were diagnosed with MASH when all three features (steatosis, lobular inflammation and ballooning) were observed in the liver biopsy

The SomaScan v.4.1 (7k) platform was used to generate SomaLogic data from serum samples. RFU computed by adaptive normalization by maximum likelihood were provided by the vendor. Samples that did not pass vendor quality control thresholds were removed. Likewise, aptamers classified as non-human or non-proteins or with median RFU below the lower limit of detection were excluded from the analysis. Differential gene expression analysis was performed using the limma v.3.52.1 R package^38^. A linear model was fit using lmFit on log_2_ RFU while adjusting for age, sex, body mass index and diabetes status. The eBayes function was used to compute moderated *t*-statistics by setting trend and robust parameters to true. CIs were computed using the limma ‘topTable’ function, setting a Bonferroni-corrected threshold of 0.05. Aptamer log_2_ fold changes and corresponding CIs were joined with the results from the mediation analysis based on SomaLogic sequence identifiers. Box plots of protein abundance across healthy and MASH samples were based on log_2_ RFU after removing effects from gender, age, diabetes and body mass index using the limma function ‘RemoveBatchEffect’.

### Semaglutide treatment in DIO-MASH and CDA-HFD mice: effects of treatment duration on liver fibrosis

The Danish Animal Experiments Inspectorate approved all experiments, which were conducted using internationally accepted principles for the use of laboratory animals (license no. 2013-15-2934-00784 and no. 2018-15-2934-00784 (Gubra) and no. 2017-15-0201-01215 (Novo Nordisk)). Each animal was identified by an implantable subcutaneous microchip (PetID Microchip; E-vet).

#### DIO-MASH studies

C57BL/6JRj mice (5–6 weeks old) were obtained from Janvier Labs and housed in a controlled environment (12-hour light/dark cycle, 21 ± 2.0 °C, humidity 50 ± 10%). Mice had ad libitum access to tap water and Gubra Amylin NASH diet (4.49 kcal g^−1^, 40 kcal % fat; of these, 46% saturated fatty acids by weight, 22% fructose, 10% sucrose, 2% cholesterol; Research Diets, D09100310) for 34 weeks for the efficacy trial. In both DIO-MASH studies, a group was maintained on regular mouse chow (2.85 kcal g^−1^; Brogaarden, Altromin 1324) for comparison. A liver biopsy was taken from the mice 4 weeks before treatment start, as described in detail previously^39–41^. In brief, for pretreatment liver biopsy, mice were anesthetized with isoflurane; a small abdominal incision in the midline was made; and the left lateral lobe of the liver was exposed. A cone-shaped wedge of liver tissue (50–100 mg) was excised from the distal part of the lobe. The cut surface of the liver was closed by electrosurgical bipolar coagulation using an electrosurgical unit (ERBE VIO 100C; ERBE). The liver was returned to the abdominal cavity; the abdominal wall was sutured; and the skin was stapled. Intraperitoneal carprofen (5 mg kg^−1^) was administered at the time of surgery and at postoperative days 1 and 2. After the procedure, animals were single-housed and allowed to recover for 4 weeks prior to treatment start. Only mice with steatosis score 3 or mice with fibrosis score >1 and steatosis score >2 were included in the efficacy trial, as outlined by Kleiner et al.^39^. Included animals were then randomized into treatment groups based on mean baseline PSR area% 1 week before dose start.

#### CDA-HFD diet study

Male C57BL/6JRj mice (7–8 weeks old) were obtained from Taconic and housed in a controlled environment (12-hour light/dark cycle, lights on at 6:00, 21 ± 1.0 °C, humidity 45–65%). Mice had ad libitum access to tap water and either chow or a high-caloric CDA-HFD diet (kcal %: fat 60%, carbohydrates 20%; 5.2 kcal g^−1^; Research Diets, A06071302) for 6 weeks prior to treatment start.

### Semaglutide treatment in DIO-MASH and CDA-HFD mice

#### Formulations

Semaglutide and vehicle were prepared at Novo Nordisk in Måløv, Denmark. Vehicle was PBS containing 0.007% polysorbate 20, 50 mM phosphate and 70 mM sodium chloride, at pH 7.4.

##### DIO-MASH study

In the efficacy study, animals were administered vehicle or semaglutide 123 µg kg^−1^ (*n* = 16) daily for 8, 16 or 24 weeks. Vehicle-dosed chow-fed mice (*n* = 10) served as additional controls.

Dosing was performed subcutaneously once daily in a volume of 5 ml kg^−1^. To reduce initial gastric discomfort, the dose was increased through daily increments until reaching the target dose on treatment day 5. The 123 µg kg^−1^ once-daily dose aimed to result in weight loss similar to that from a clinical dose of 2.4 mg/weekly, with adjustments for species differences in half-life. Body weight was monitored daily during the intervention period.

##### CDA-HFD study

CDA-HFD mice were randomly allocated to groups and treated with vehicle or semaglutide 20 µg kg^−1^ (*n* = 15) for 6 weeks or 12 weeks. A group of CDA-HFD mice was euthanized at treatment start to determine baseline levels of MASH (*n* = 10, baseline group). Vehicle-dosed chow-fed mice (*n* = 5) served as additional controls. Body weight was monitored every 3 days during the intervention period.

### Sampling and histology

For histological analysis, a full-thickness slab of the left lateral lobe was fixed in 10% neutral buffered formalin and routine processed to paraffin blocks.

### Liver histology and image analysis

Paraffin-embedded liver tissue was sectioned (nominal 4-µm thickness) and mounted on SuperFrost Plus slides. Sections of liver tissue were stained with hematoxylin and eosin (H&E), PSR, anti-αSMA (Abcam, ab124964 (0.4 µg ml^−1^) and ab5694 (0.2 µg ml^−1^)) or anti-type I collagen (Southern Biotech; Col1a1, 1310-01 (4 µg ml^−1^)) using standard procedures^40^. Quantitative histomorphometry was applied using digital imaging software (Visiomorph, Visiopharm). Fractional (%) area of liver fat (macrosteatosis) was determined on H&E-stained sections. PSR, αSMA and Col1a1 immunostaining was expressed as a fraction of the total parenchymal area without steatosis by subtracting the fraction of fat area determined on adjacent H&E-stained sections.

### Next-generation RNA sequencing

All groups finishing the treatment phase were included in RNA sequencing analysis. Liver samples of 20 ± 10 mg were taken from the left lateral lobe, snap frozen in liquid nitrogen and stored at −70 °C. RNA was purified using a NucleoSpin Kit (Macherey-Nagel). Purified RNA (10 ng to 1 µg) from each sample was used to generate a cDNA library using an NEBNext Ultra II Directional RNA Library Prep Kit for Illumina. The cDNA library was then sequenced on a NextSeq 500 using NextSeq 500/550 High Output Kit V2 (Illumina).

The sequencing data were aligned to the mm10 (GRCm38) transcriptome, obtained from the Ensembl database, using STAR v.2.7.3a. Read counts were quantified by salmon v.1.2.0; read quality of the data was evaluated using FastQC 0.11.9 and Picard; and the intergroup and intragroup variability was evaluated using principal component analysis and hierarchical clustering. Differential gene expression was assessed by the R package DESeq2.

### GLP-1 receptor expression in mouse and human liver

#### Human liver biopsies

Twenty-six human diagnostic, formalin-fixed, paraffin-embedded histological liver needle biopsies were retrieved from the archives at Aalborg Hospital in Denmark. The study was conducted in accordance with the ethical standards of the 1964 Declaration of Helsinki and its later amendments. It was checked that the participants had not stated in the Tissue Application Register (Vævsanvendelsesregisteret) at the Danish Data Protection Agency that biobanked material must not be used for research. The biopsies were fully anonymized, and it was not possible to identify the donors of the biobanked material.

GLP-1 receptor expression was assessed in human liver biopsies as described previously^42,43^ with some modifications. In short, the sections were microwave-treated in TEG buffer (pH 9.0) (Ampliqon) for 15 minutes and allowed to cool for 15 minutes. Slides were rinsed in tap water and treated with 1% H_2_O_2_ in Tris-buffered saline for 15 minutes and rinsed in Tris-buffered saline followed by avidin/biotin blocking. The sections were pre-incubated with 0.5% TNB blocking buffer (PerkinElmer) for 2 hours and incubated with the validated GLP-1 receptor monoclonal antibody (Mab 3F52) at 5 µg ml^−1^ (ref. ^43^) overnight at 4 °C in 0.5% TNB blocking buffer. The next day, sections were incubated with VECTASTAIN ABC (Vector Laboratories) and developed with DAB+ (Enzo Life Sciences). Slides were washed with Tris-buffered saline with 0.05% Tween between incubations.

In situ hybridization was performed on the human liver biopsies on a Ventana Discovery Ultra automation system (Ventana Medical Systems) using an RNAscope VS Universal HRP/AP Kit (Advanced Cell Diagnostics) and the specific GLP1R probe, RNAscope 2.5 VS Probe-Hs-GLP1R (Advanced Cell Diagnostics, cat. no. 519829). Positive (peptidylprolyl isomerase B, NM_011149.2) and negative (Bacillus Subtillis, dihydropicolinate reductase, EF191515) in situ hybridization control probes were employed for assay validation in all analyses.

#### Mouse liver

In mice, GLP-1 receptor expression was assessed using immunohistochemistry in three liver lobes from each of one chow/vehicle dosed and four DIO-NASH vehicle or CDA-HFD vehicle mice. The tissues were analyzed on the Ventana Discovery Ultra automation system (Ventana Medical Systems) for GLP-1 receptor expression using rabbit-anti-mGLP1R (Abcam, ab218532, lot: GR3231665-2) at a concentration of 2.7 µg ml^−1^. In brief, 5-µm sections were baked at 60 °C for 32 minutes and then deparaffinized at 72 °C for 24 minutes. Pretreatment in buffer CC1 was at 95 °C for 16 minutes, followed by incubation in HRP block for 12 minutes. After application of primary antibody, slides were incubated at 37 °C for 60 minutes and then detected with anti-rabbit HQ at 35 °C for 24 minutes, followed by anti-HQ HRP 35 °C for 16 minutes. Chromogen (Purple) was applied for 32 minutes, and then sections were counterstained with Hematoxylin II for 8 minutes and with bluing reagent for 4 minutes. Pancreas, duodenum, stomach and kidney were used as positive control tissues using the same automated immunohistochemistry protocol.

#### Statistics and reproducibility

The phase 2 clinical trial was powered to show a difference in proportions of 28% on primary endpoint MASH resolution and no worsening of fibrosis between once-daily semaglutide 0.4 mg (assumed 45% response) and placebo (assumed 17% response). The study was randomized (1:1:1:1: placebo; semaglutide 0.1 mg; semaglutide 0.2 mg; semaglutide 0.4 mg), and investigators and patients were blinded. For the proteomics analysis, no formal power calculation was conducted. However, all statistical proteomics analyses were rigorously adjusted for multiple testing using the FDR procedure. No data were excluded. For the mediation analysis on the histology endpoints evaluating change in body weight as mediator, data were based on complete-case on-treatment measurements to evaluate the mechanistic action of semaglutide in MASH (hence, data outside the on-treatment observation period were excluded). The estimated effects are presented along with 95% CIs (that is, not adjusted for multiplicity). The preclinical studies were powered based on power calculations on the endpoints with highest variability (alanine aminotransferase and Col1 area %) in previous studies. DIO-MASH animals with steatosis score ≥2 and a fibrosis stage of ≥1, based on liver biopsy taken 4 weeks before treatment start, were included. Animals were randomized into treatment groups on percentage fractional area of fibrosis (PSR staining) in the pretreatment biopsy. CDA-HFD animals were allocated to treatment groups without randomization. An age-matched control group on regular chow diet was included, without randomization. Data from animals that did not finish the study were excluded.

### Reporting summary

Further information on research design is available in the Nature Portfolio Reporting Summary linked to this article.

## Online content

Any methods, additional references, Nature Portfolio reporting summaries, source data, extended data, supplementary information, acknowledgements, peer review information; details of author contributions and competing interests; and statements of data and code availability are available at https://doi.org/10.1038/s41591-025-03799-0.

## Supplementary information

Supplementary Table 1 SomaSignalNASH tests—proteins assessed.

Reporting Summary

## Source data

Source Data Fig. 1 Statistical source data.

Source Data Fig. 2 Statistical source data.

Source Data Fig. 3 Statistical source data.

Source Data Fig. 4 Statistical source data. Note that source data for **a** cannot be shared as they comprise confidential patient-level data.

Source Data Fig. 5 Statistical source data.

Source Data Table 1 Statistical source data.

Source Data Extended Data Fig. 1 Statistical source data.

Source Data Extended Data Fig. 2 Statistical source data.

Source Data Extended Data Fig. 3 Statistical source data.

Source Data Extended Data Fig. 4 Statistical source data.

Source Data Extended Data Fig. 6 Immunohistochemistry and in situ hybridization original images—provided as a multi-page PDF.

Source Data Extended Data Fig. 7 Statistical source data.

Source Data Extended Data Fig. 8 Statistical source data.

## Figures and Tables

**Extended Data Table 1:**
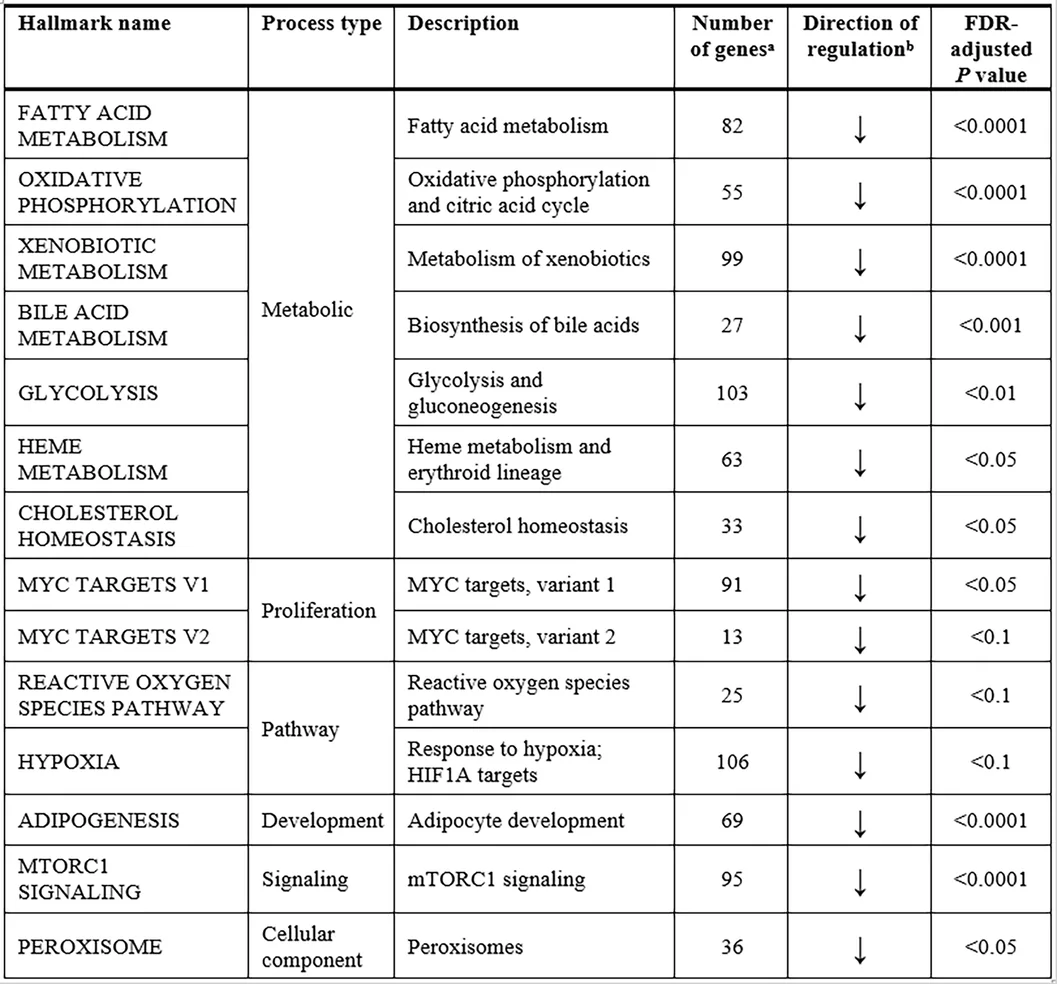
Effects of semaglutide versus placebo on 14 hallmark gene sets in individuals with MASH receiving once-daily subcutaneous semaglutide 0.4 mg or placebo for 72 weeks

A gene set enrichment analysis was performed using the hallmark gene set collection of 50 gene sets generated from the Molecular Signatures Database. Model estimates (from a mixed model for repeated measures analysis) for the treatment ratio of semaglutide 0.4 mg/placebo at week 72 for all 4,979 protein biomarkers in the SomaScan assay were included in the analysis. Statistically significant effect of semaglutide 0.4 mg versus placebo was defined as an FDR-adjusted *P* value (*q* value) < 0.2. Only gene sets with significant treatment effect of semaglutide 0.4 mg versus placebo are shown. As an example, the top-ranked enriched hallmark gene set was fatty acid metabolism. The table shows that the gene set is downregulated, meaning that genes/proteins in this pathway are expressed less in patients treated with high-dose semaglutide compared to placebo.

^a^Included in the analysis

^b^Semaglutide 0.4 mg versus placebo.

HIF1A, hypoxia-inducible factor 1-alpha; MASH, metabolic dysfunction-associated steatohepatitis; mTORC1, mammalian target of rapamycin complex 1.

## Data Availability

Access request proposals can be found at https://www.novonordisk-trials.com/. Data must not be used for commercial purposes. RNA sequencing data obtained from the DIO-MASH and CDA-HFD animal studies will be publicly available in the Gene Expression Omnibus (https://www.ncbi.nlm.nih.gov/geo/) under their respective data repository accession numbers: GSE294629 and GSE294630. Details regarding the hallmark gene set collection are provided in ref. ^12^. Data are available at https://www.gsea-msigdb.org/gsea/msigdb/. Source data are provided with this paper.

## Code availability

Code for preprocessing SomaScan proteomics data, performing statistical analyses and generating figures is available via Zenodo at https://doi.org/10.5281/zenodo.13356055 (ref. ^44^). R v.4.3.1 is available at https://cran.r-project.org/bin/windows/base/old/4.3.1/.
