# Supplementary material for: Modulation of metabolic, inflammatory and fibrotic pathways by semaglutide in metabolic dysfunction-associated steatohepatitis
Source: Nat Med. 2025 Jul 21;31(9):3128–40. doi: 10.1038/s41591-025-03799-0 (PMC12443624; doi:10.1038/s41591-025-03799-0)
Supplement: Supplementary file 1 — SomaSignalNASH tests—proteins assessed. [file 41591_2025_3799_MOESM1_ESM.pdf]

nature medicine

Supplementary information

https://doi.org/10.1038/s41591-025-03799-0

Modulation of metabolic, inflammatory and fibrotic pathways by semaglutide in metabolic dysfunction-associated steatohepatitis

In the format provided by the authors and unedited

Supplementary Table 1. SomaSignal® NASH tests – proteins assessed.

SomaSignal NASH test	Protein marker
Steatosis	Insulin-like peptide INSL5 (INSL5)<br>Fatty acid-binding protein 12 (FABP12)<br>Atp-dependent dna helicase q1 (RECQL)<br>Beta-glucuronidase (GUSB)<br>Inhibin beta c chain (INHBC)<br>Beta-hexosaminidase subunit beta (HEXB)<br>Beta-ala-his dipeptidase (CNDP1)<br>Growth hormone variant (GH2)<br>Prostaglandin reductase 1 (PTGR1)<br>Bpi fold-containing family b member 1 (BPIFB1)<br>Glutamate receptor ionotropic delta-2 (GRID2)<br>Serine/threonine-protein kinase/endoribonuclease ire1 (ERN1)
Inflammation	Aminoacylase-1(ACY1)<br>Dolichyl-diphospho-oligosaccharide protein glycosyltransferase subunit 1 (RPN1)<br>Uncharacterized protein c1orf198 (C1orf198)<br>Transcriptional repressor ctcf (CTCF)<br>Serum amyloid a-2 protein (SAA2)<br>Low-affinity immunoglobulin gamma fc region receptor iii-b (FCGR3B)<br>Adiponectin (ADIPOQ)<br>Thioredoxin reductase 1 (TXNRD1)<br>Maleylacetoacetate isomerase (GSTZ1)<br>Tumor-associated calcium signal transducer 2 (TACSTD2)<br>Peptide yy (PYY)<br>c-c motif chemokine 23 (CCL23)<br>Procollagen c-endopeptidase enhancer 2 (PCOLCE2)<br>Low molecular weight phosphotyrosine protein phosphatase (ACP1)
Ballooning	Ado-keto reductase family 1 member b10 (AKR1B10)

**SomaSignal NASH test**	**Protein marker**
	Prostaglandin reductase 1 (PTGR1)<br>Adamts-like protein 2 (ADAMTSL2)<br>Cytotoxic t-lymphocyte protein 4 (CTLA4)<br>Calponin-2 (CNN2)
Fibrosis	Adamts-like protein 2 (ADAMTSL2)<br>Complement component c7 (C7)<br>Neurofascin (NFASC)<br>Collectin-11 (COLEC11)<br>Vascular endothelial growth factor receptor (KDR)<br>Protein wnt-5 (WNT5A)<br>Procollagen-lysine 2-oxoglutarate 5-dioxygenase 3 (PLOD3)<br>fc receptor-like protein 3 (FCRL3)
