# Supplementary material for: Modulation of metabolic, inflammatory and fibrotic pathways by semaglutide in metabolic dysfunction-associated steatohepatitis
Source: Nat Med. 2025 Jul 21;31(9):3128–40. doi: 10.1038/s41591-025-03799-0 (PMC12443624; doi:10.1038/s41591-025-03799-0)
Supplement: Supplementary file 2 — Reporting Summary [file 41591_2025_3799_MOESM2_ESM.pdf]

nature portfolio

Corresponding author(s): Lotte Bjerre Knudsen

Last updated by author(s): May 14, 2025

Reporting Summary

Nature Portfolio wishes to improve the reproducibility of the work that we publish. This form provides structure for consistency and transparency in reporting. For further information on Nature Portfolio policies, see our Editorial Policies and the Editorial Policy Checklist.

Statistics

For all statistical analyses, confirm that the following items are present in the figure legend, table legend, main text, or Methods section.

n/a	Confirmed	
☐	☒	The exact sample size (*n*) for each experimental group/condition, given as a discrete number and unit of measurement
☐	☒	A statement on whether measurements were taken from distinct samples or whether the same sample was measured repeatedly
☐	☒	The statistical test(s) used AND whether they are one- or two-sided<br>*Only common tests should be described solely by name; describe more complex techniques in the Methods section.*
☐	☒	A description of all covariates tested
☐	☒	A description of any assumptions or corrections, such as tests of normality and adjustment for multiple comparisons
☐	☒	A full description of the statistical parameters including central tendency (e.g. means) or other basic estimates (e.g. regression coefficient) AND variation (e.g. standard deviation) or associated estimates of uncertainty (e.g. confidence intervals)
☐	☒	For null hypothesis testing, the test statistic (e.g. *F*, *t*, *r*) with confidence intervals, effect sizes, degrees of freedom and *P* value noted<br>*Give P values as exact values whenever suitable.*
☒	☐	For Bayesian analysis, information on the choice of priors and Markov chain Monte Carlo settings
☒	☐	For hierarchical and complex designs, identification of the appropriate level for tests and full reporting of outcomes
☒	☐	Estimates of effect sizes (e.g. Cohen's *d*, Pearson's *r*), indicating how they were calculated

Our web collection on statistics for biologists contains articles on many of the points above.

Software and code

Policy information about availability of computer code

Data collection	Code for preprocessing SomaScan® proteomics data, performing statistical analyses and generating figures is available via Zenodo at https://doi.org/10.5281/zenodo.13356055
Data analysis	Statistical mediation analysis was done using the R package: R v4.3.1 is available at https://cran.r-project.org/bin/windows/base/old/4.3.1/

For manuscripts utilizing custom algorithms or software that are central to the research but not yet described in published literature, software must be made available to editors and reviewers. We strongly encourage code deposition in a community repository (e.g. GitHub). See the Nature Portfolio guidelines for submitting code & software for further information.

Data

Policy information about availability of data

All manuscripts must include a data availability statement. This statement should provide the following information, where applicable:

- Accession codes, unique identifiers, or web links for publicly available datasets
- A description of any restrictions on data availability
- For clinical datasets or third party data, please ensure that the statement adheres to our policy

Access request proposals can be found at https://www.novonordisk-trials.com/. Data must not be used for commercial purposes. RNA sequencing data from the animal studies will be publicly available in the Gene Expression Omnibus (https://www.ncbi.nlm.nih.gov/geo/) under data repository accession numbers GSE294629

(DIO-MASH) and GSE294630 (CDA HFD). Details regarding the hallmark gene set collection are provided in ref. 12. Data are available at https://www.gsea-msigdb.org/gsea/msigdb/.

Research involving human participants, their data, or biological material

Policy information about studies with human participants or human data. See also policy information about sex, gender (identity/presentation), and sexual orientation and race, ethnicity and racism.

Reporting on sex and gender	The proteomics analyses in this phase 2 semgalutide trial were not stratified by gender due to the limited sample size of the phase 2 study. In a previous publication (Maretty L, et al. Proteomic changes upon treatment with semaglutide in individuals with obesity. Nature Medicine 31;267–277 (2025)) in the much larger phase 3 STEP trial of semaglutide for weight management, we found no evidence that gender modified the effect of semaglutide.
Reporting on race, ethnicity, or other socially relevant groupings	Not applicable
Population characteristics	Among participants in the phase 2 trial, 61% (n = 193) were women, 78% (n = 248) were White, and 13% (n = 40) were Hispanic or Latino. Mean participant age was 55 years, mean body weight was 98.4 kg, and mean body mass index was 35.8 kg/m2. 165 out of 320 randomized patients (52%) had a body mass index of ≥35.
Recruitment	Reported in previously published phase 2b study (Newsome PN, et al. N Engl J Med 2021;384:1113-1124)
Ethics oversight	The protocol was approved by the institutional review board and ethics committee at each participating trial site. The protocol was made available for the following publication;<br>Newsome PN, et al. N Engl J Med 2021;384:1113-1124 and can be downloaded here https://www.nejm.org/doi/full/10.1056/NEJMoa2028395#ap1

Note that full information on the approval of the study protocol must also be provided in the manuscript.

Field-specific reporting

Please select the one below that is the best fit for your research. If you are not sure, read the appropriate sections before making your selection.

☒ Life sciences ☐ Behavioural & social sciences ☐ Ecological, evolutionary & environmental sciences

For a reference copy of the document with all sections, see nature.com/documents/nr-reporting-summary-flat.pdf

Life sciences study design

All studies must disclose on these points even when the disclosure is negative.

Sample size	320 adult participants were included in the phase 2b clinical trial. For SomaScan® proteomic profiling and SomaSignal® NASH tests, 1,088 serum samples were included representing 293 of the enrolled 320 subjects.
Data exclusions	For some participants, samples were not shipped to SomaLogic® for analysis due to lack of consent or availability of samples in the storage facility upon the cut-off date for the use of samples. Following pre-processing and quality control of the SomaLogic® data, a total of 10 serum samples failed.
Replication	No explicit replication of the presented findings was performed. However, SomaScan® proteomic data were obtained from a subset of participants in the Copenhagen Cohort of MASLD (CoCoMASLD, formerly known as FLINC [Fatty Liver Disease in Nordic Countries] cohort; ClinicalTrials.gov: NCT04340817, H-17029039) whose data sets included clinical measurements and archived samples. SomaScan® data analysis of 235 participants were available: 146 with MASH and 89 healthy volunteers.
Randomization	Samples were derived from a phase 2b clinical trial in which participants were randomized to receive semaglutide at doses of 0.1 mg, 0.2 mg or 0.4 mg or placebo via once-weekly subcutaneous injection for 72 weeks. Please refer to the original publication for further details on randomization (Newsome PN, et al. N Engl J Med 2021;384:1113-1124).
Blinding	The phase 2b clinical trial was double blinded. Please refer to the original publication for further details on blinding (Newsome PN, et al. N Engl J Med 2021;384:1113-1124). In the current work, adaptive normalization by maximum likelihood was used for the SomaScan® proteomic data as recommended by SomaLogic. Relative fluorescence units (RFU) from each sample were normalized based on hybridization controls on each microarray to correct for systematic variability in hybridization, and median signal based on all features for each dilution to correct for variability across plates according to the SomaScan® Data Standardization guidelines (SomaScan® Data Standardization and File Specification Technical Note [SSM-020]). All RFUs were natural logarithm transformed for uniformity.

Reporting for specific materials, systems and methods

We require information from authors about some types of materials, experimental systems and methods used in many studies. Here, indicate whether each material, system or method listed is relevant to your study. If you are not sure if a list item applies to your research, read the appropriate section before selecting a response.

Materials & experimental systems

n/a	Involved in the study	
☐	☒	Antibodies
☒	☐	Eukaryotic cell lines
☒	☐	Palaeontology and archaeology
☐	☒	Animals and other organisms
☐	☒	Clinical data
☒	☐	Dual use research of concern
☒	☐	Plants

Methods

n/a	Involved in the study	
☒	☐	ChIP-seq
☒	☐	Flow cytometry
☒	☐	MRI-based neuroimaging

Antibodies

Antibodies used	Anti-αSMA (#ab124964 [0.4 µg/ml]; #ab5694 [0.2 µg/ml], Abcam, Cambridge, UK), or anti-type I collagen (Col1a1, #1310-01[4 µg/ml], Southern Biotech, Birmingham, AL, USA), Abcam ab218532 rabbit-anti-mGLP1R, lot: GR3231665-2 at a concentration of 2.7 ug/ml
Validation	Validation citation: Pyke, C., et al. GLP-1 receptor localization in monkey and human tissue: novel distribution revealed with extensively validated monoclonal antibody. Endocrinology 155, 1280–1290 (2014)

Animals and other research organisms

Policy information about studies involving animals; ARRIVE guidelines recommended for reporting animal research, and Sex and Gender in Research

Laboratory animals	CDA HFD mice were 9-10 weeks on initiation of dietary induction and 15-16 weeks old at the start of treatment. DIO-MASH animals were 5-6 weeks at the start of diet intervention and 34-35 weeks old at the start of treatment.
Wild animals	No wild animals were used
Reporting on sex	The study used only male mice. We have performed similar experiments with female mice, and they reproduce the disease phenotype to a slightly milder degree and show a similar response to treatment.
Field-collected samples	No field collected samples were used
Ethics oversight	Danish Animal Experiments Inspectorate

Note that full information on the approval of the study protocol must also be provided in the manuscript.

Clinical data

Policy information about clinical studies

All manuscripts should comply with the ICMJE guidelines for publication of clinical research and a completed CONSORT checklist must be included with all submissions.

Clinical trial registration	Phase 2b study - NCT02970942
Study protocol	The protocol was made available for the following publication; Newsome PN, et al. N Engl J Med 2021;384:1113-1124 and can be downloaded here https://www.nejm.org/doi/full/10.1056/NEJMoa2028395#ap1
Data collection	With respect to data collection from human biosamples, the study protocol states; "The samples will be stored at a bio-repository after end of trial and until marketing authorisation approval or until the research project terminates, but no longer than 15 years from end of trial after which they will be destroyed
Outcomes	Primary endpoint: > NASH resolution without worsening of fibrosis after 72 weeks. Resolution of NASH was defined by comprehensive interpretation by two independent pathologists (central reading) blinded to treatment allocation and with complete resolution captured by terms such as “no fatty liver disease” or “simple steatosis or isolated steatosis” and defined by the NASH Clinical research network (CRN) as “no more than mild residual inflammatory cells and no ballooning”. Worsening of fibrosis was defined as an increase of at least one stage of the Kleiner fibrosis classification Supportive secondary efficacy endpoints were; 1) at least one stage of liver fibrosis improvement with no worsening of NASH after 72 weeks (worsening defined as an increase of at least one stage of either lobular inflammation or hepatocyte ballooning according to NASH CRN criteria); 2) Change from baseline to week 72 in NAFLD activity score (NAS); 3) Change from baseline to week 72 in Fibrosis-4 score, ALT, AST and GGT.

Plants

Seed stocks	*Report on the source of all seed stocks or other plant material used. If applicable, state the seed stock centre and catalogue number. If plant specimens were collected from the field, describe the collection location, date and sampling procedures.*
Novel plant genotypes	*Describe the methods by which all novel plant genotypes were produced. This includes those generated by transgenic approaches, gene editing, chemical/radiation-based mutagenesis and hybridization. For transgenic lines, describe the transformation method, the number of independent lines analyzed and the generation upon which experiments were performed. For gene-edited lines, describe the editor used, the endogenous sequence targeted for editing, the targeting guide RNA sequence (if applicable) and how the editor was applied.*
Authentication	*Describe any authentication procedures for each seed stock used or novel genotype generated. Describe any experiments used to assess the effect of a mutation and, where applicable, how potential secondary effects (e.g. second site T-DNA insertions, mosiacism, off-target gene editing) were examined.*
