# Supplementary material for: Modulation of metabolic, inflammatory and fibrotic pathways by semaglutide in metabolic dysfunction-associated steatohepatitis
Source: Nat Med. 2025 Jul 21;31(9):3128–40. doi: 10.1038/s41591-025-03799-0 (PMC12443624; doi:10.1038/s41591-025-03799-0)

Extended data figure 6. Panel a source images.

Positive control tissues from C57Bl/6 normal mouse.

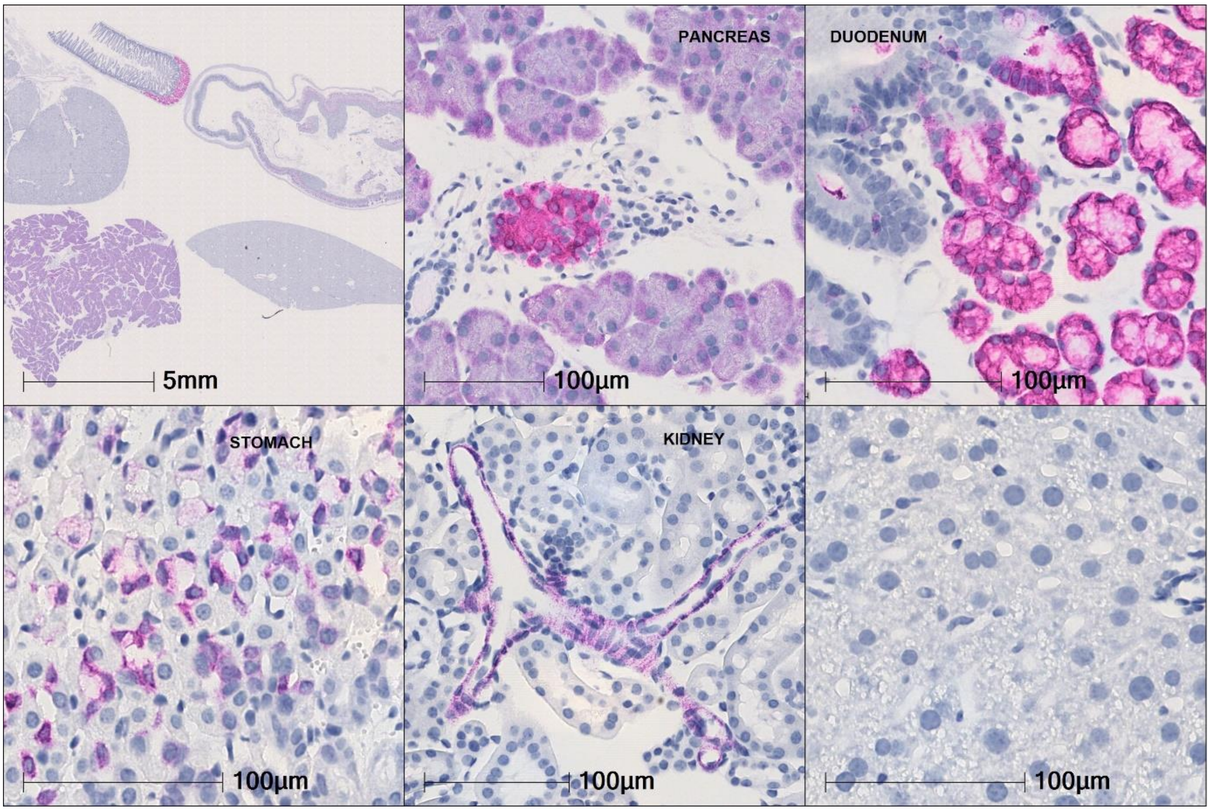

Livers from DIO-NASH and CDA-HFD mice.

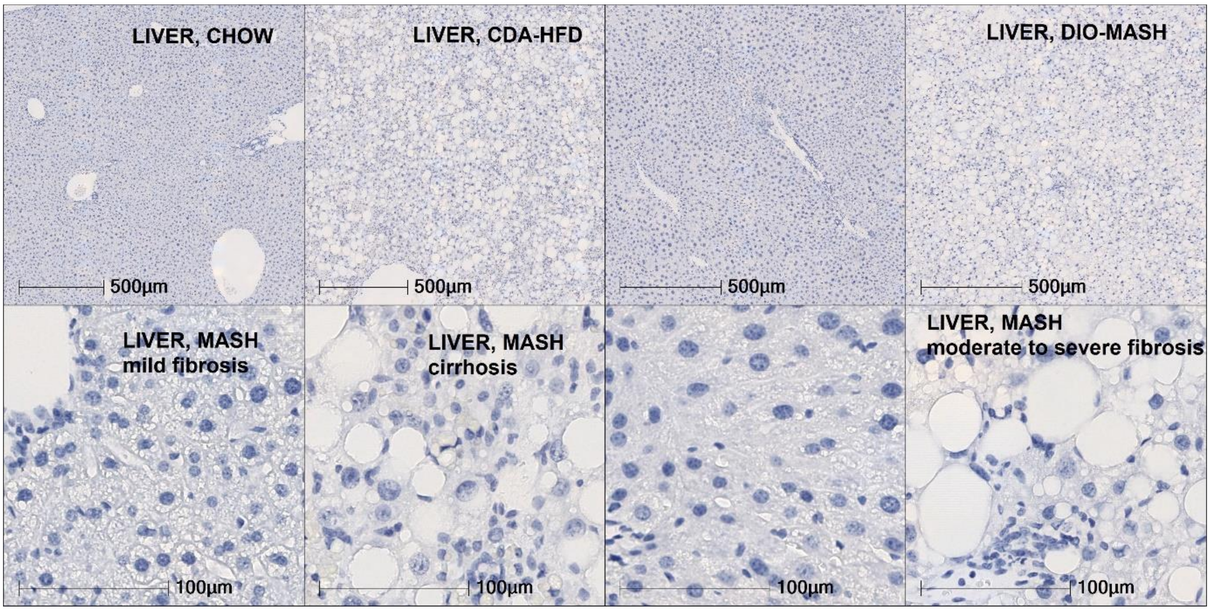

Panel b source images. Assessment of GLP-1 receptor mRNA expression in human liver by RNAscope in situ hybridization.

Pancreas

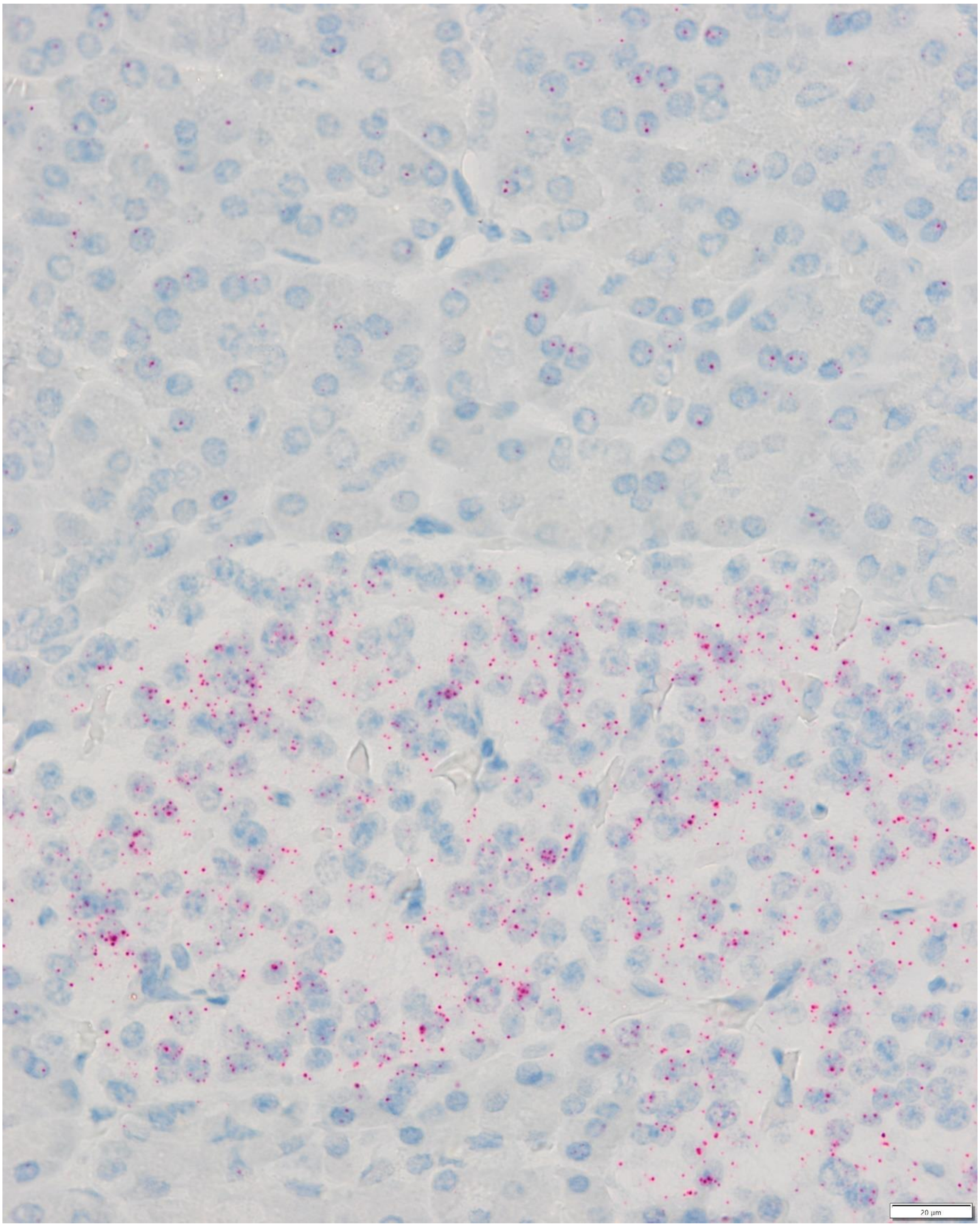

Liver, normal

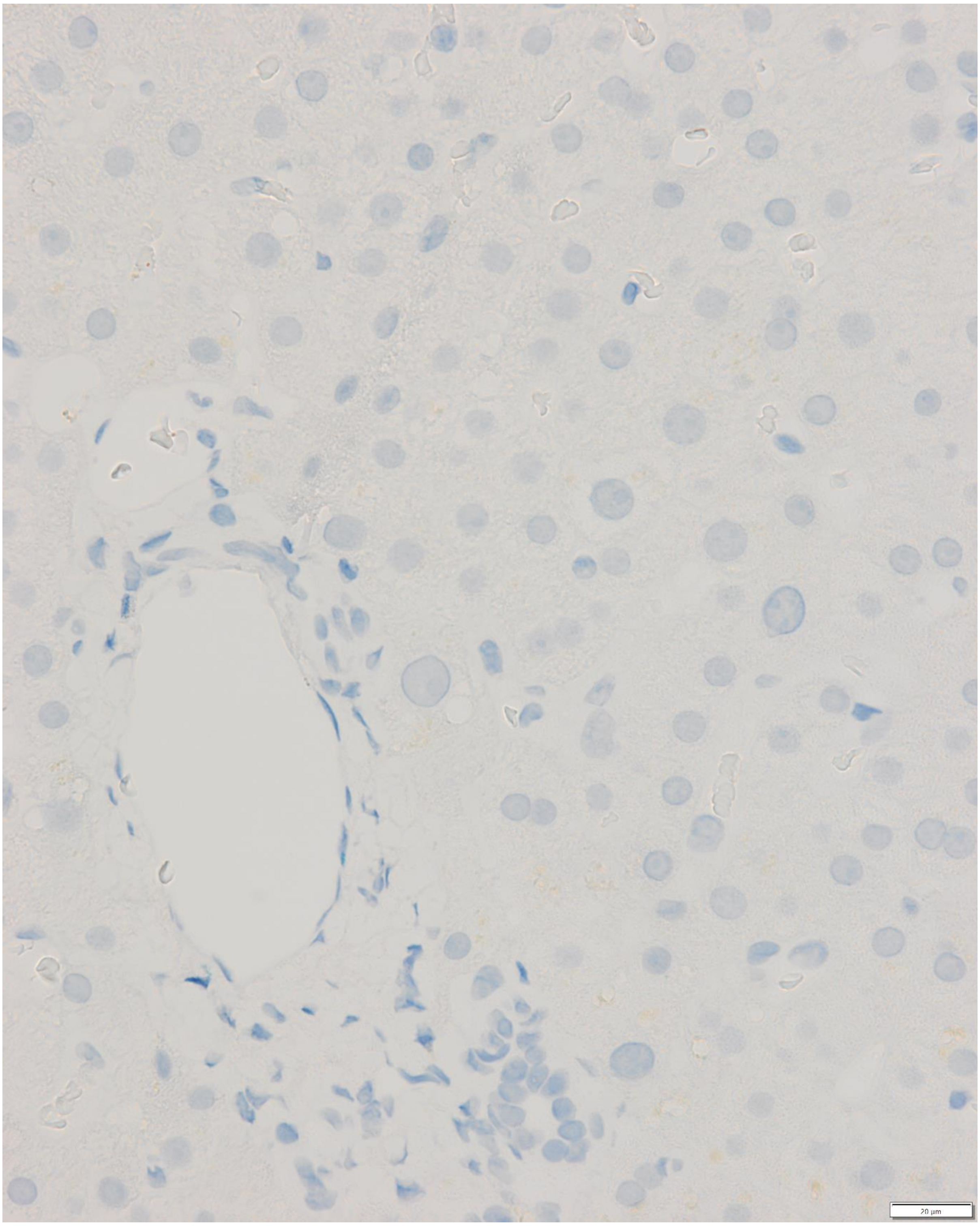

Liver. MASH, mild fibrosis

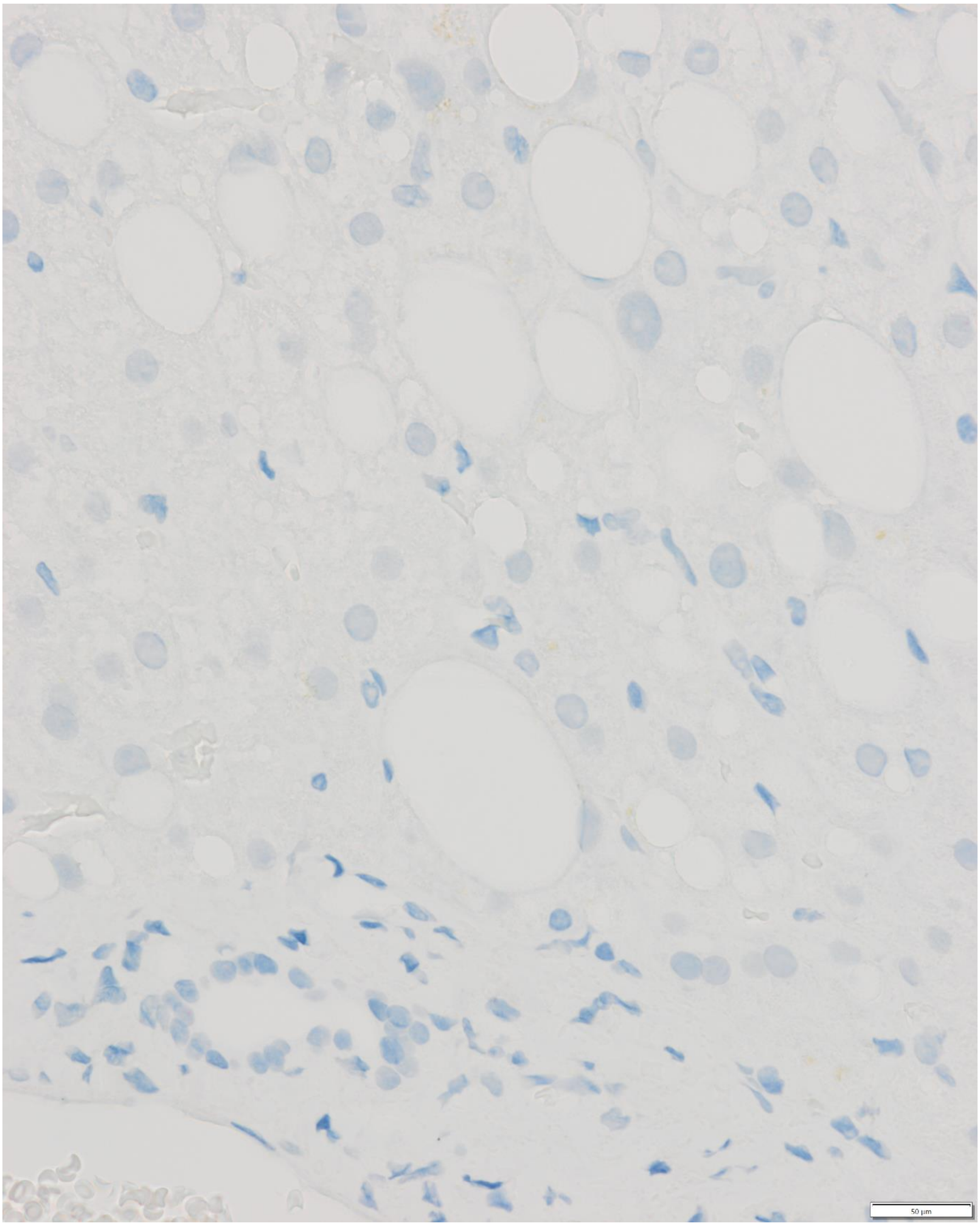

Liver. MASH, moderate to severe fibrosis

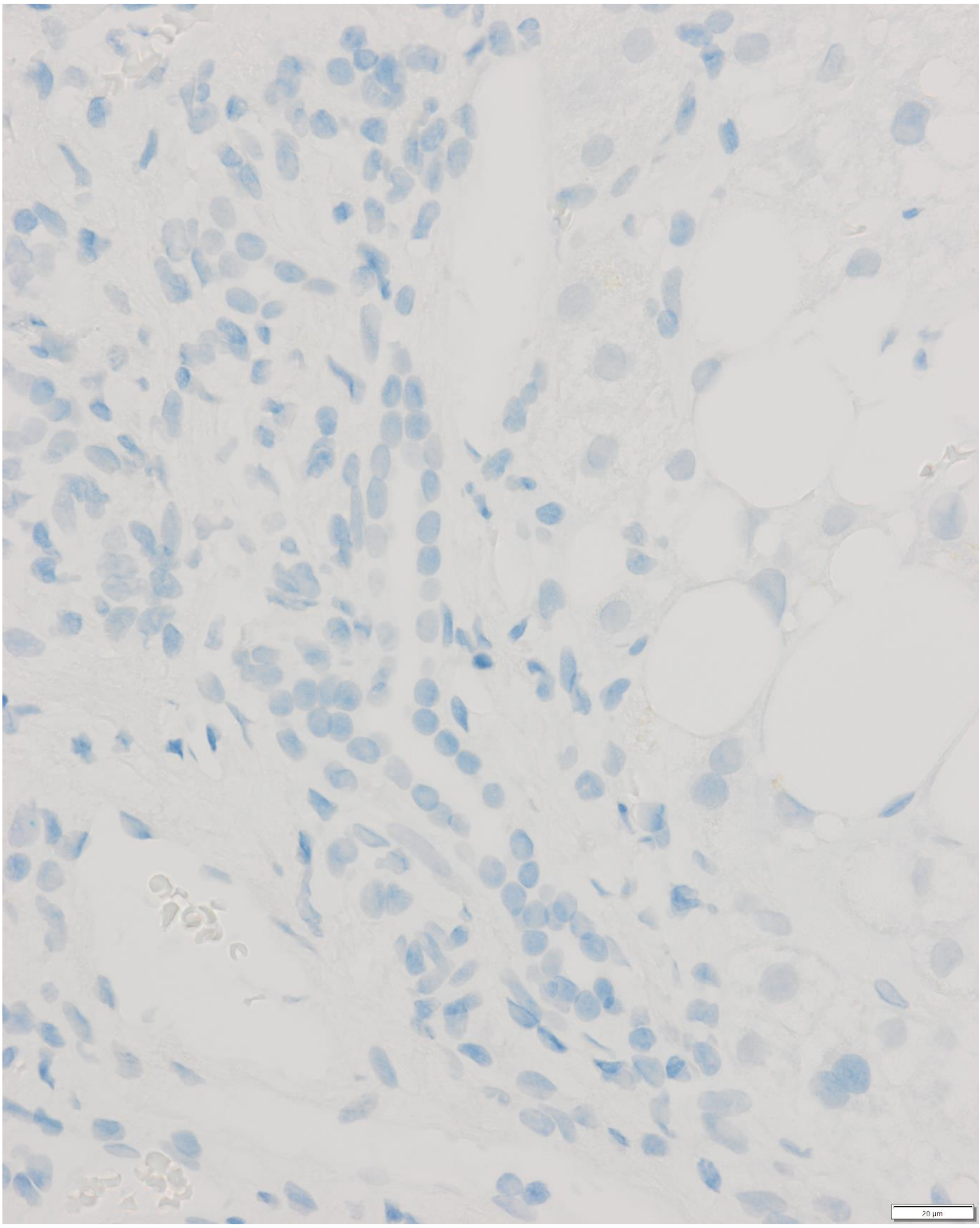

Livee, MASH, cirrhosis

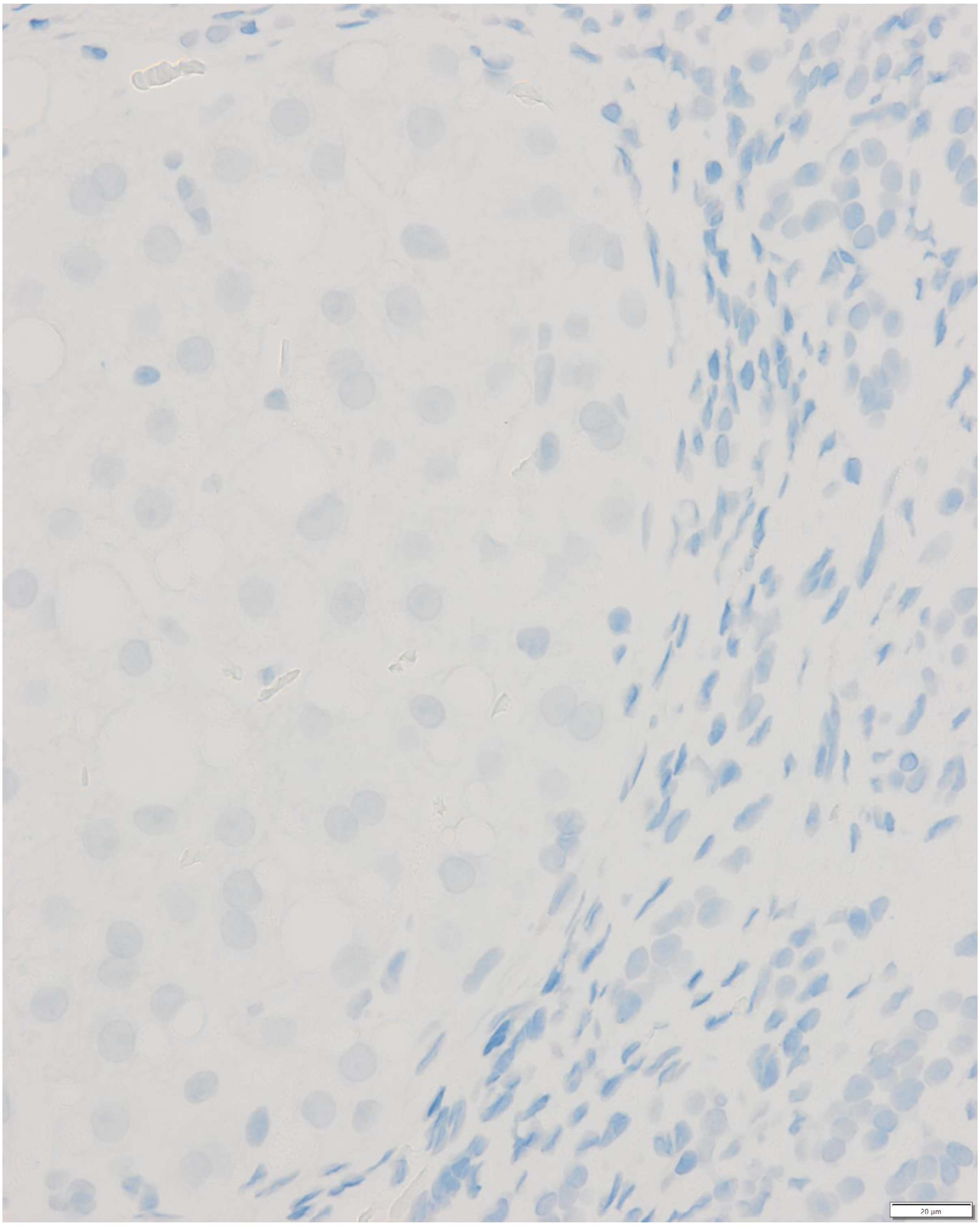

Panel b source images. Assessment of GLP-1 receptor protein expression in human liver by immunohistochemistry.

Pancreas

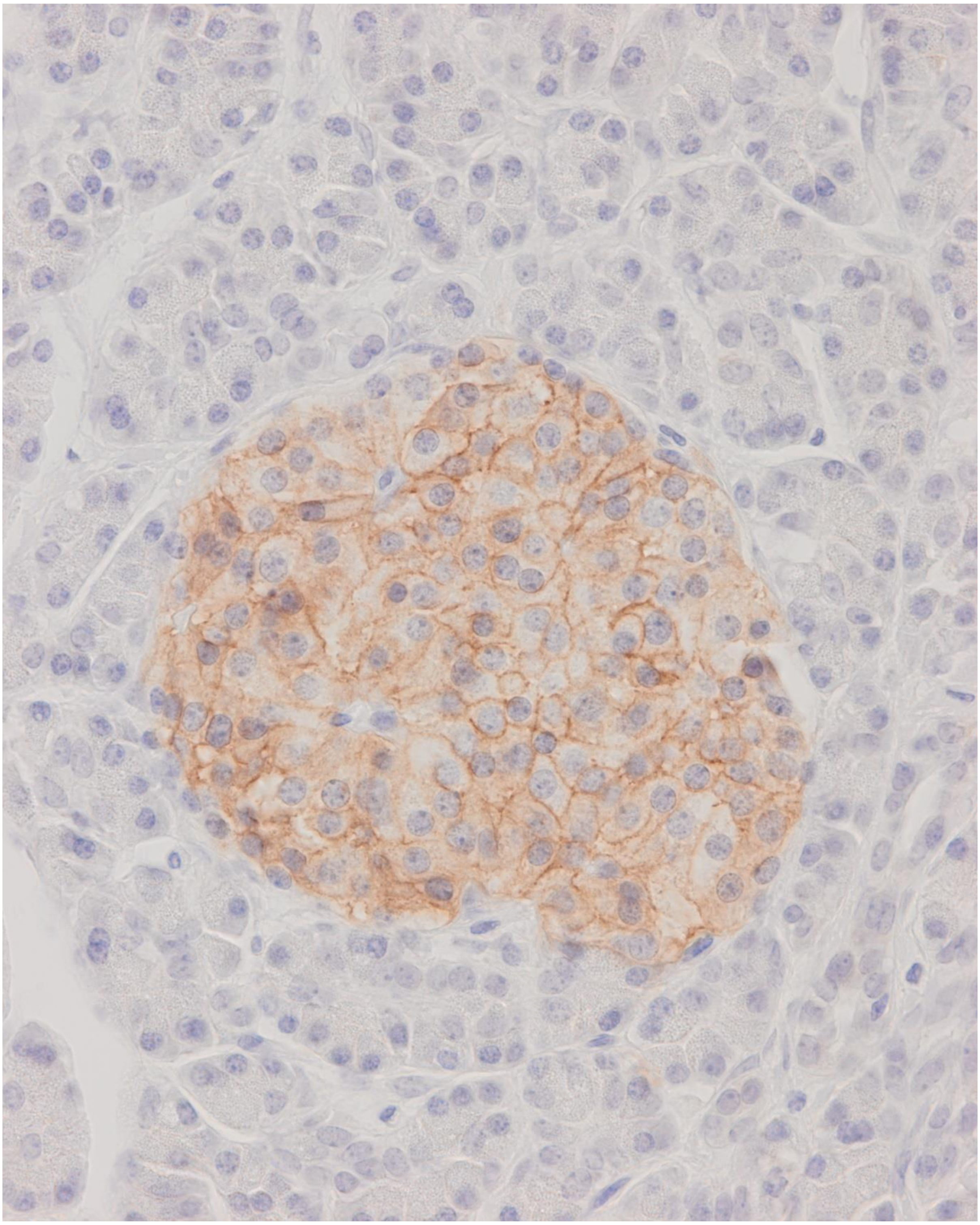

Liver, normal

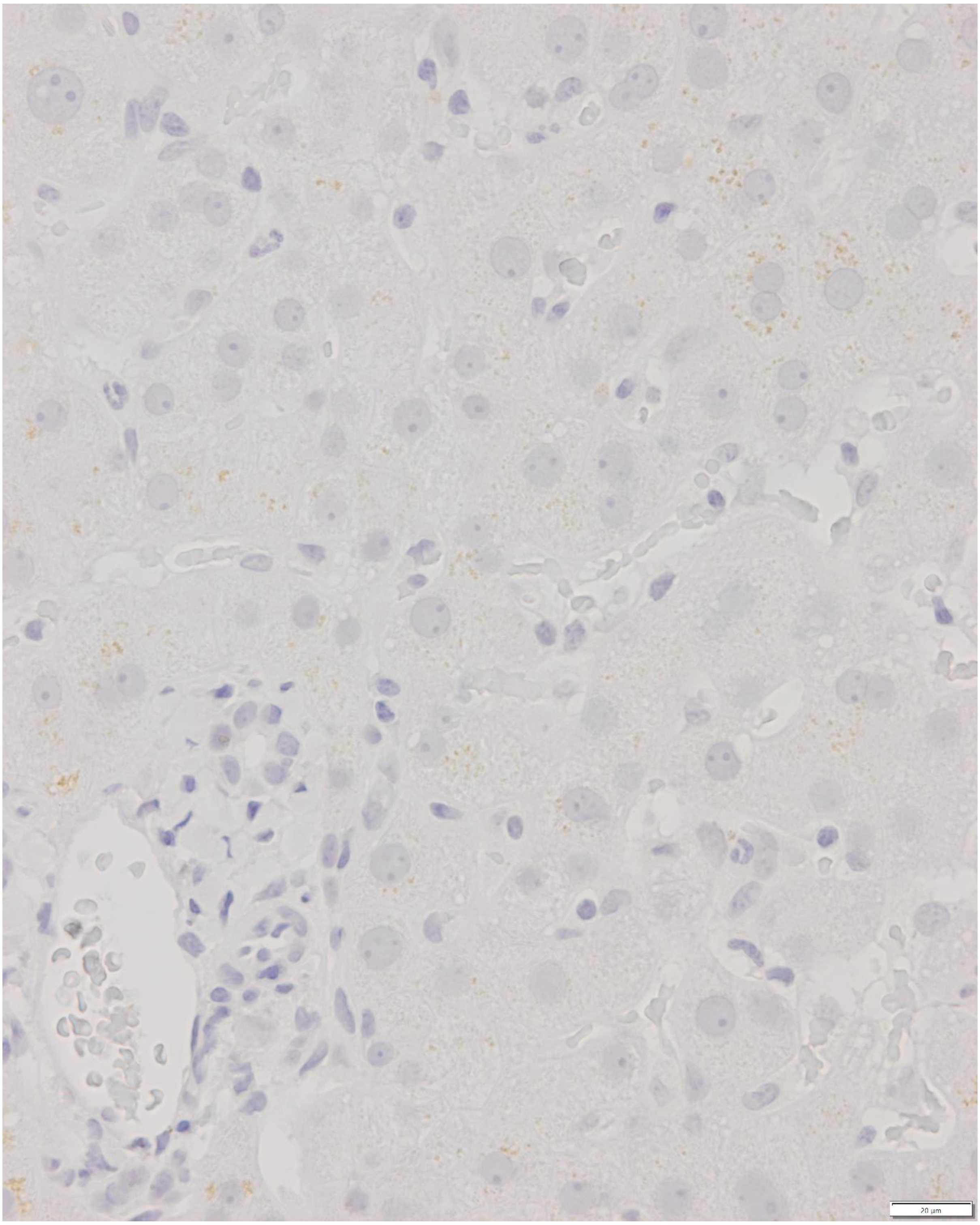

Liver, MASH, mild fibrosis

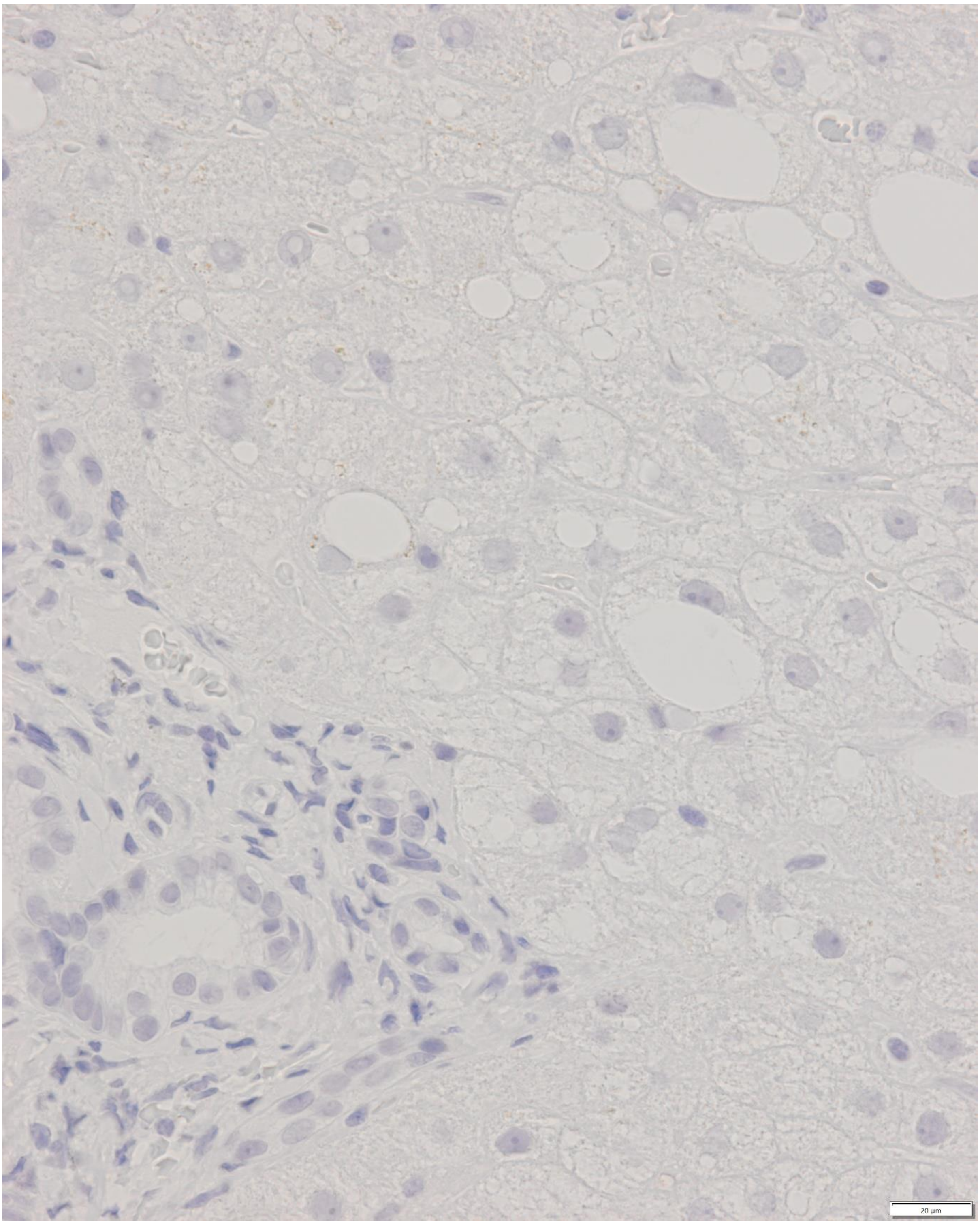

Liver. MASH, moderate to severe fibrosis

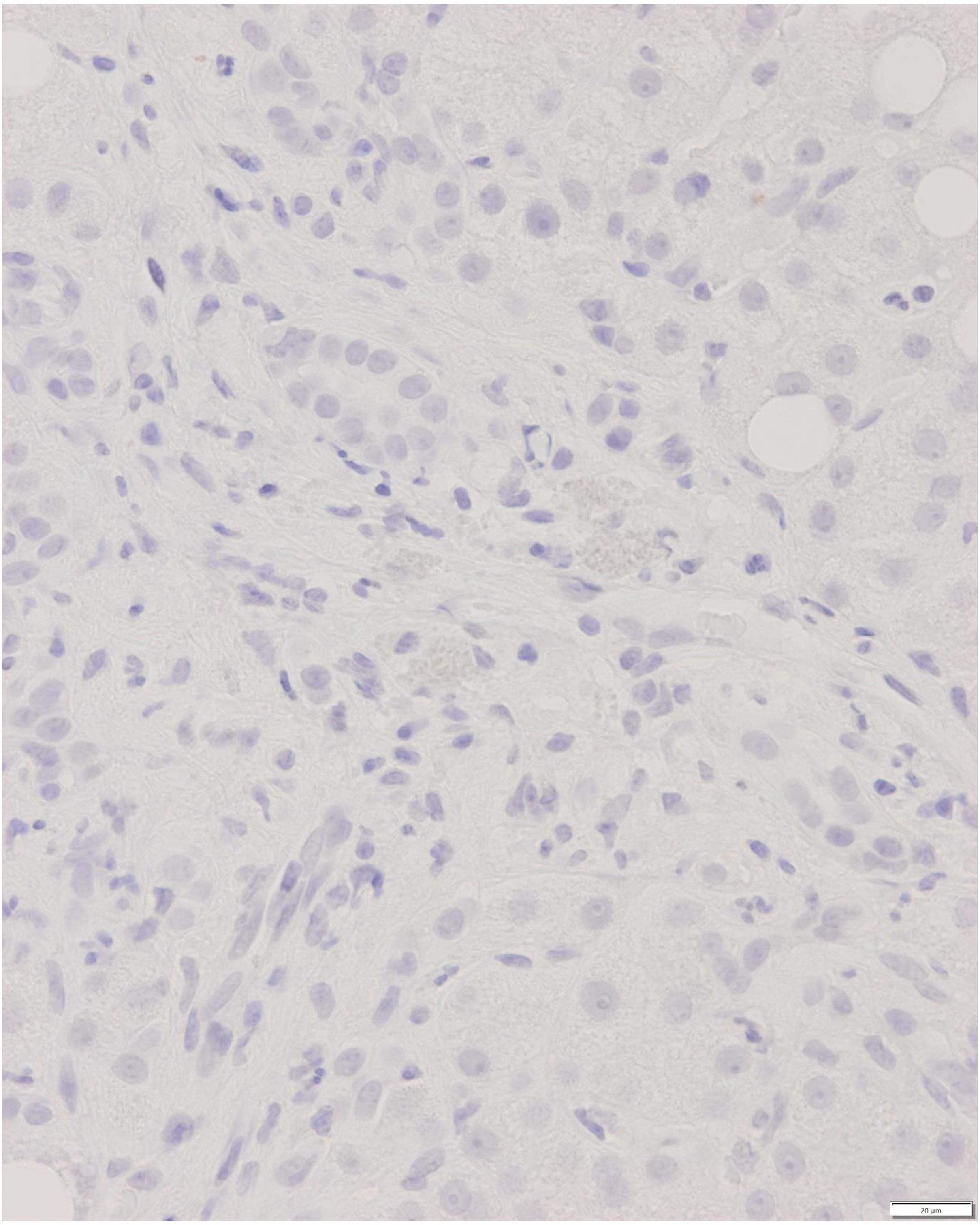

Liver, MASH, cirrhosis

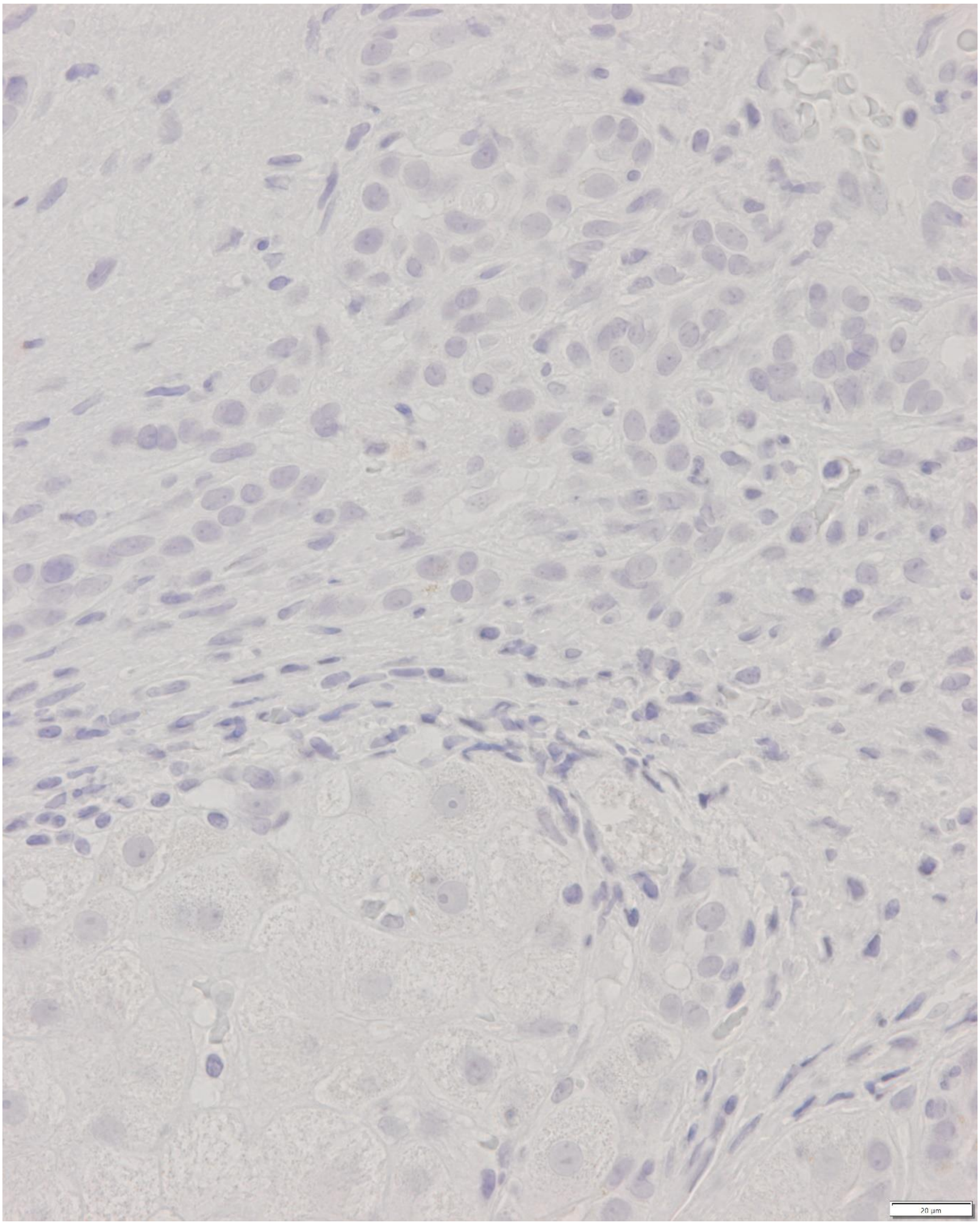

Supplement: Supplementary file 13 — Immunohistochemistry and in situ hybridization original images—provided as a multi-page PDF. [file 41591_2025_3799_MOESM13_ESM.pdf]
